# COVID-19 vaccination boosts the potency and breadth of the immune response against SARS-CoV-2 among recovered patients in Wuhan

**DOI:** 10.1038/s41421-022-00496-x

**Published:** 2022-12-09

**Authors:** Hong Liang, Xuanxuan Nian, Junzheng Wu, Dong Liu, Lu Feng, Jia Lu, Yan Peng, Zhijun Zhou, Tao Deng, Jing Liu, Deming Ji, Ran Qiu, Lianzhen Lin, Yan Zeng, Fei Xia, Yong Hu, Taojing Li, Kai Duan, Xinguo Li, Zejun Wang, Yong Zhang, Hang Zhang, Chen Zhu, Shang Wang, Xiao Wu, Xiang Wang, Yuwei Li, Shihe Huang, Min Mao, Huanhuan Guo, Yunkai Yang, Rui Jia, Jingwei Xufang, Xuewei Wang, Shuyan Liang, Zhixin Qiu, Juan Zhang, Yaling Ding, Chunyan Li, Jin Zhang, Daoxing Fu, Yanlin He, Dongbo Zhou, Cesheng Li, Jiayou Zhang, Ding Yu, Xiao-Ming Yang

**Affiliations:** 1 Beijing Tiantan Biological Products Co., Ltd., Beijing, China; 2 National Engineering Technology Research Center for Combined Vaccines, Wuhan, Hubei China; 3 Wuhan Institute of Biological Products Co., Ltd., Wuhan, Hubei China; 4 Chengdu Rongsheng Pharmaceuticals Co., Ltd., Chengdu, Sichuan China; 5 Sinopharm Wuhan Plasma-derived Biotherapies Co., Ltd., Wuhan, Hubei China; 6 Wuxue Wusheng Plasma Collection Center, Wuxue, Hubei China; 7 China National Biotec Group Company Limited, Beijing, China; 8 Wuhan Biobank Co., Ltd., Wuhan, Hubei China

**Keywords:** Innate immunity, Cell biology, Autoimmunity

## Abstract

The immunity of patients who recover from coronavirus disease 2019 (COVID-19) could be long lasting but persist at a lower level. Thus, recovered patients still need to be vaccinated to prevent reinfection by severe acute respiratory syndrome coronavirus 2 (SARS-CoV-2) or its mutated variants. Here, we report that the inactivated COVID-19 vaccine can stimulate immunity in recovered patients to maintain high levels of anti-receptor-binding domain (RBD) and anti-nucleocapsid protein (NP) antibody titers within 9 months, and high neutralizing activity against the prototype, Delta, and Omicron strains was observed. Nevertheless, the antibody response decreased over time, and the Omicron variant exhibited more pronounced resistance to neutralization than the prototype and Delta strains. Moreover, the intensity of the SARS-CoV-2-specific CD4^+^ T cell response was also increased in recovered patients who received COVID-19 vaccines. Overall, the repeated antigen exposure provided by inactivated COVID-19 vaccination greatly boosted both the potency and breadth of the humoral and cellular immune responses against SARS-CoV-2, effectively protecting recovered individuals from reinfection by circulating SARS-CoV-2 and its variants.

## Introduction

Coronavirus disease 2019 (COVID-19) is caused by severe acute respiratory syndrome coronavirus-2 (SARS-CoV-2) and continues to spread globally, leading to substantial mortality and morbidity^1^. The emergence of extensively mutated SARS-CoV-2 variants, such as Alpha (B.1.1.7), Beta (B.1.351), Gamma (P.1), Kappa (B.1.617.1), Delta (B.1.617.2), and Omicron (B.1.1.529), has raised concerns about resistance to neutralizing antibodies (NAbs) generated during previous infections, thereby making the prevention and control of the COVID-19 pandemic more difficult. Omicron BA.1 is a transient lineage that was rapidly replaced by the Omicron sublineage BA.2^2^. The antibodies that cross-react to SARS-CoV-2 variants are very important for preventing reinfection by future strains. A more worrisome consequence is that individuals who previously recovered from COVID-19 can be reinfected. A study from South Africa reported that the Omicron variant may evade immunity derived from the previous infection in recovered patients and is more likely than other variants to reinfect people^3^.

The SARS-CoV-2-specific immune response mediated by both antibodies and T cells plays a crucial role in maintaining long-term immunity against SARS-CoV-2. There have been many studies on humoral and cellular immunity after primary infection. SARS-CoV-2-specific antibodies are essential to prevent viral reinfection, but the antibody responses from the initial wave of infections waned rapidly over a period of months after COVID-19 patients recovered^4–7^. Anamnestic recall of memory T cell populations can also prevent infection or disease from re-exposure^8^. Although SARS-CoV-2-specific T cell immunity has been reported to be retained for more than 6 months or even 12 months^9,10^, the latest research suggested that the functions of SARS-CoV-2-specific CD4^+^ T cells may be exhausted 1 year after recovery^11^. Decreased humoral and cellular immunity in recovered patients possibly leads to an increase in breakthrough infections a year or even months after recovery. Hence, for individuals with or without a history of infection, vaccination is the most crucial strategy against COVID-19.

Globally, several safe and effective COVID-19 vaccines have been applied, which can decrease the risk of SARS-CoV-2 infection and the burden of COVID-19 on public health, society, and the economy worldwide, including Sinopharm WIBP-CorV, Sinopharm BBIBP-CorV, Sinovac CoronaVac, Janssen Ad26.COV2. S, Moderna mRNA-1273, Zhifei ZF2001, and Pfizer BNT162b2^12–18^. The spike (S) protein of SARS-CoV-2 can bind to human angiotensin-converting enzyme 2 (hACE2) via its receptor-binding domain (RBD), therefore allowing SARS-CoV-2 to enter cells^19^. The N-terminal domain (NTD) also plays a pivotal role in the fusion transition of S protein^20^. Thus, both the RBD and NTD of S protein are frequently selected as critical epitopes for NAbs, and the efficacy of most mRNA (mRNA-1273, BNT162b2), protein subunit (ZF2001) or adenovirus-vectored (Ad26.COV2.S) vaccines against COVID-19 are dependent on anti-SARS-CoV-2 S or RBD neutralization activity. Similar to vaccines for poliovirus, Japanese encephalitis virus, hepatitis A virus vaccines, etc., COVID-19 inactivated vaccines maintain a high degree of integrity for recognition by the human immune system^16,21,22^. The inactivated vaccines include the S protein, nucleocapsid protein (NP), and other structural proteins of SARS-CoV-2. The non-neutralizing antibodies against structural proteins generated by such vaccination may activate diverse effector functions regulated by their Fc domain^23^.

COVID-19 mRNA and inactivated vaccines are currently the most widely used vaccine types in the world during the COVID-19 pandemic. Studies have shown that mRNA vaccination of recovered patients strongly enhances the breadth and potency of the humoral immune response against SARS-CoV-2^24,25^. However, there are almost no studies related to inactivated vaccines, and it remains unknown how inactivated vaccines affect humoral and cellular immune responses in recovered patients. Therefore, this study aimed to elucidate the persistence and breadth of antibody responses as well as CD4^+^ T cell responses against SARS-CoV-2 and its mutants in recovered patients after vaccination.

## Results

### Participant cohort and study design

**Fig. 1:**
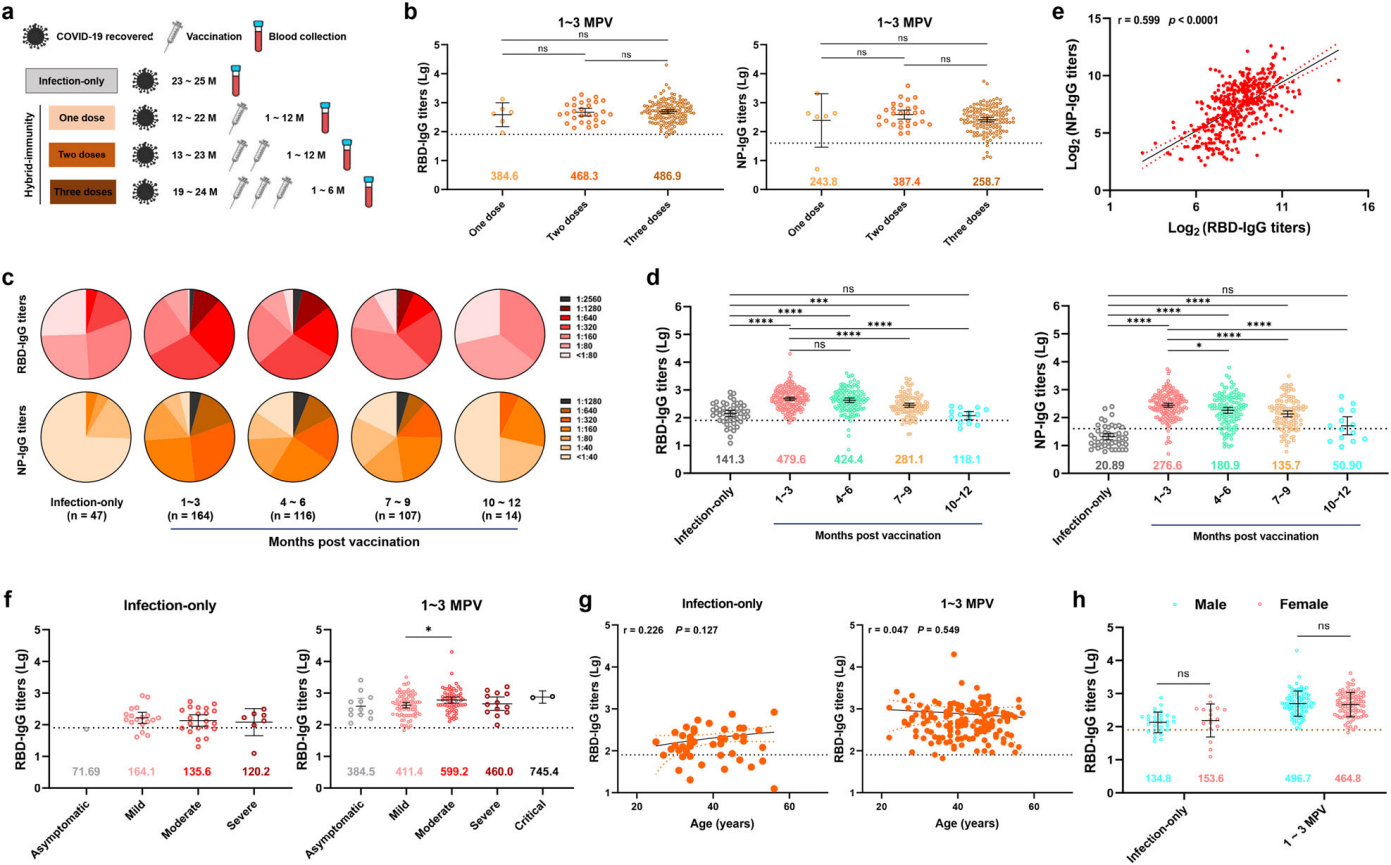
RBD-IgG and NP-IgG levels in unvaccinated or vaccinated individuals who previously recovered from COVID-19. **a** Schematic diagram representing the sequential order and time scale of the natural infection and vaccination groups. The black virus particle denotes recovery from COVID-19, the gray syringe represents a dose of vaccine, and the blue-capped vial represents blood sample collection. Months were calculated based on the time of the final dose of vaccination. **b** Comparison of the RBD-IgG and NP-IgG titers of patients receiving one, two, or three vaccine doses in the hybrid-immunity group at 1–3 months post vaccination. **c** Distribution of RBD-IgG and NP-IgG titers in the infection-only and hybrid-immunity groups. Changes in the distribution of RBD-IgG and NP-IgG titers at 1–3, 4–6, 7–9, and 10–12 months are shown, and darker colors indicate higher titers. **d** Alteration of RBD-IgG and NP-IgG titer responses over time in the hybrid-immunity group, with the infection-only group serving as a control. **e** RBD-IgG titers were positively correlated with NP-IgG titers (*n* = 448). **f**–**h** No correlation between RBD-IgG titers and disease severity (**f**), age (**g**) or sex (**h**) was detected. Error bars in **b**, **d**, **f**, and **h** show the GMT values with 95% confidence intervals, and the colored numbers in the figures represent GMT values. Statistical significance in **b**, **d**, and **f** was analyzed using the Kruskal–Wallis test. Only groups with significant differences are marked in **f**. Statistical significance in **h** was analyzed using the paired *t*-test. Scatter plots in **e** and **g** denote the simple linear fit with 95% confidence bands, together with the Spearman correlation coefficient and 2-tailed *P* value. M, months; MPV, months post vaccination.

A total of 448 COVID-19 participants in Wuhan were recruited (Table 1), including 47 recovered patients who had not been vaccinated (infection-only) and 401 recovered patients who had been vaccinated (hybrid-immunity). The group design and sample collection information are shown in Fig. 1a. All participants were recovered patients previously infected with the SARS-CoV-2 prototype strain and were not reinfected. The hybrid-immunity group consisted of recovered patients who received different numbers of vaccine doses, including 105 individuals with one dose, 160 individuals with two doses, and 136 individuals with three doses. A total of 833 doses of inactivated COVID-19 vaccines, including WIBP-CorV (360, 43.22%), BBIBP-CorV (142, 17.05%), CoronaVac (307, 36.85%), and KCONVAC^26^ (24, 2.88%), were used to vaccinate 401 recovered patients. These four inactivated vaccines were all developed based on the SARS-CoV-2 prototype strain, sharing similar production techniques in which all the SARS-CoV-2 vaccine strains were cultured in Vero cells, inactivated by β-propiolactone, and mixed with an aluminum hydroxide adjuvant^16,27–29^.

**Table 1:** Cohort demographics.

Characteristic	Infection-only (*n*)	Hybrid-immunity (*n*)		
		One dose	Two doses	Three doses
***Donors***	47	105	160	136
***Sex***				
Male	30	64	86	71
Female	17	41	74	65
***Age, years*** **(mean** ± **SD)**	39.38 ± 8.12	40.48 ± 7.55	40.24 ± 8.62	41.98 ± 8.00
***Months post vaccination***^a^				
1–3	NA	6	28	130
4–6	NA	32	78	6
7–9	NA	56	51	0
10–12	NA	11	3	0
***Disease severity***				
Asymptomatic	1	8	16	9
Mild	18	46	74	63
Moderate	21	43	59	53
Severe	7	7	11	9
Critical	0	1	0	2

*NA* not applicable.

^a^Months were calculated after the final dose of vaccination.

Serum specimens were collected to examine the presence and titers of immunoglobulin G (IgG) antibodies against the RBD (RBD-IgG) and NP (NP-IgG) of the SARS-CoV-2 prototype strain by enzyme-linked immunosorbent assay (ELISA). Next, the average 50% inhibitory concentration (IC_50_) neutralization titer values were assessed by using both pseudotyped and authentic virus-based assays. Peripheral blood mononuclear cell (PBMC) samples were used for intracellular cytokine staining (ICS) and activation-induced marker (AIM) assays.

### COVID-19 vaccination restores the specific IgG response against SARS-CoV-2 in recovered patients

The ELISA geometric mean titer (GMT) values were determined within 3 months post vaccination for RBD-IgG and NP-IgG levels in the recovered patients who received one, two or three vaccine doses, and the results demonstrated that there was no obvious difference among these three groups (Fig. 1b). Consistently, the neutralization assay based on both pseudotyped and authentic viruses also revealed no remarkable difference among these three groups (Supplementary Fig. S1). Consequently, these three groups of vaccinated recovered patients were combined into a single group (hereafter referred to as the hybrid-immunity group). Our findings are consistent with studies of humoral immune responses in recovered patients after receipt of different doses of mRNA vaccine^24,30^.

For RBD-IgG, titers of ≥ 1:2560 and 1:1280 were considered high titers, 1:320–1:640 were considered moderate titers, 1:80–1:160 were considered low titers, and less than 1:80 were considered negative, which is in accordance with the thresholds in our previous study^7^. The negative rate of RBD-IgG in the infection-only group was 25.5% at 23–25 months post recovery, while only 25.5% of the NP-IgG titers were 1:40 or higher (Fig. 1c), indicating the variability of RBD-IgG and NP-IgG antibody responses. For both RBD-IgG and NP-IgG titers, the proportion of higher titers decreased in a time-dependent manner, while that of lower titers exhibited the opposite trend over time (Fig. 1c), indicating that the humoral response diminished in these recovered patients. Supporting the efficiency of vaccines, vaccination enhanced RBD-IgG and NP-IgG titers, with the GMT values increased by 3.4-fold and 13.2-fold, respectively, within 3 months post vaccination compared with those of the infection-only group, while high antibody titers were maintained for 6 months (Fig. 1d). However, both RBD-IgG and NP-IgG titers decreased significantly 7–9 months post vaccination and were no longer significantly different compared with those of the infection-only group at 10–12 months post vaccination (Fig. 1d). Regardless of receipt of one, two or three vaccine doses, the GMT values of the two SARS-CoV-2-specific antibodies showed a time-dependent decreasing trend in recovered patients (Supplementary Fig. S2a, b). Additionally, there were obvious variations in RBD-IgG titer levels among individuals after vaccination, which may be due to the different potencies of individual immune responses; thus, characterization based on a large sample size could reduce such variation and lead to more conclusive results.

In addition, the correlation between the RBD-IgG and NP-IgG titers was measured using the serum samples of 448 COVID-19 participants, and the results showed that RBD-IgG titers were positively correlated with NP-IgG titers (Fig. 1e). Moreover, this correlation was repeatedly observed across populations with different disease severities (Supplementary Fig. S2c). The RBD-IgG titers in the infection-only and hybrid-immunity groups indicated that there was no correlation of antibody response with disease severity (Fig. 1f), age (Fig. 1g) or sex (Fig. 1h). Additionally, this lack of correlation was also apparent for NP-IgG titers (Supplementary Fig. S2d–f).

### Serum antibodies from vaccinated patients are capable of neutralizing pseudotyped SARS-CoV-2

The original Omicron (the BA.1 sublineage) variant rapidly replaced Delta a few weeks after it was first discovered but was soon replaced by Omicron BA.2 and BA.5, which have become the dominant variants worldwide^2,31^. Next, vesicular stomatitis virus-based SARS-CoV-2 pseudotyped viruses were employed to calculate the half-maximal IC_50_ values of neutralizing titers in the infection-only (*n* = 23) and hybrid-immunity (*n* = 105) serum samples. In the 4 time periods (1–3, 4–6, 7–9, and 10–12 months post vaccination), the samples of the hybrid-immunity group were selected according to the ratio of RBD-IgG negative, low, moderate, and high titers (Fig. 1c, up), and samples from patients vaccinated with different doses were included (Supplementary Table S1).

**Fig. 2:**
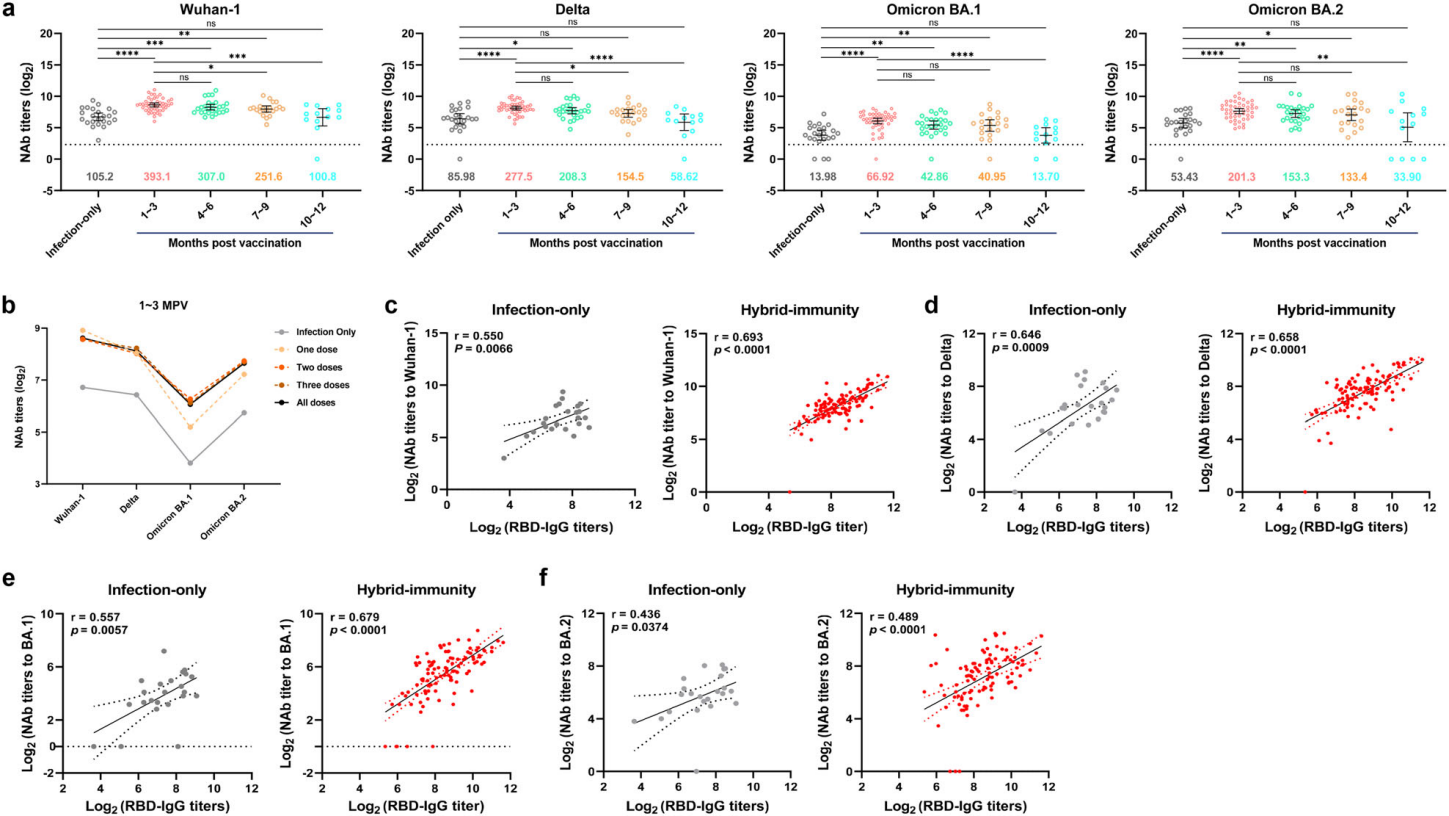
Antiviral activity of serum antibodies against pseudotyped SARS-CoV-2. **a** NAb titers against the pseudotyped SARS-CoV-2 Wuhan-1, Delta, Omicron BA.1 and BA.2 strains in the hybrid-immunity group at 1–3, 4–6, 7–9, and 10–12 months post vaccination, with the infection-only group serving as a control. The dotted line represents the limit of detection (1:5); below the limit of detection, values were set to 1:1. Error bars represent the GMT values (displayed as colored numbers in the figure) with a 95% confidence interval. Statistical significance was analyzed using the Kruskal–Wallis test. **b** Serum neutralizing activity measured as the GMT against the pseudotyped SARS-CoV-2 in the infection-only group and hybrid-immunity group (one, two, three, and all doses at 1–3 months post vaccination). **c**–**f** The correlations between NAb titers (GMT) and Wuhan-1 (**c**), Delta (**d**), Omicron BA.1 (**e**), BA.2 (**f**) and RBD-IgG titers in the infection-only (left) and hybrid-immunity (right) groups. Scatter plots depict the simple linear fit with 95% confidence bands, together with the Spearman correlation coefficient and 2-tailed *P* value. Wuhan-1, SARS-CoV-2 Wuhan-Hu-1; MPV, months post vaccination.

The GMT values of NAbs for SARS-CoV-2 were markedly higher in the hybrid-immunity group than in the infection-only group for SARS-CoV-2 Wuhan-Hu-1 (Wuhan-1), Delta, Omicron BA.1, and Omicron BA.2 strains and persisted up to 7–9 months post vaccination (Fig. 2a). Regardless of one, two or three doses, the GMT values of NAbs for these pseudotyped SARS-CoV-2 all showed a time-dependent decline in recovered patients who were vaccinated (Supplementary Fig. S3). The GMT values of NAbs in the hybrid-immunity group within 3 months post vaccination were 3.7-, 3.2-, 4.8- and 3.8-fold higher than those in the infection-only group for the Wuhan-1, Delta, Omicron BA.1 and Omicron BA.2 strains, respectively (Fig. 2b).

It was found that for all four pseudotyped SARS-CoV-2 strains, the neutralizing titers were associated with RBD-IgG levels in both the infection-only and hybrid-immunity groups. In addition, the hybrid-immunity group was more strongly correlated with the Wuhan-1, Delta, Omicron BA.1 and BA.2 strains, and the hybrid-immunity group had a smaller *P* value (Fig. 2c–f). Our results showed that the quality and breadth of humoral immunity could be improved after inoculation with inactivated vaccines.

### Vaccination regenerates neutralizing activity against SARS-CoV-2 in recovered COVID-19 patients

To further understand the potency and durability of the serum antibody response among the infection-only and hybrid-immunity groups, the authentic SARS-CoV-2 nCoV-2019BetaCoV/Wuhan/WIV04/2019 (WIV04), Delta, Omicron BA.1, BA.2 and BA.5 strains were used to monitor the neutralizing titers with cell-level microneutralization assays. Additionally, the IC_50_ values of neutralizing titers in infection-only (*n* = 11) and hybrid-immunity (*n* = 89) serum samples were computed accordingly. The sample selection was based on criteria similar to that for the sample selection for assays with pseudotyped SARS-CoV-2 (Supplementary Table S1).

**Fig. 3:**
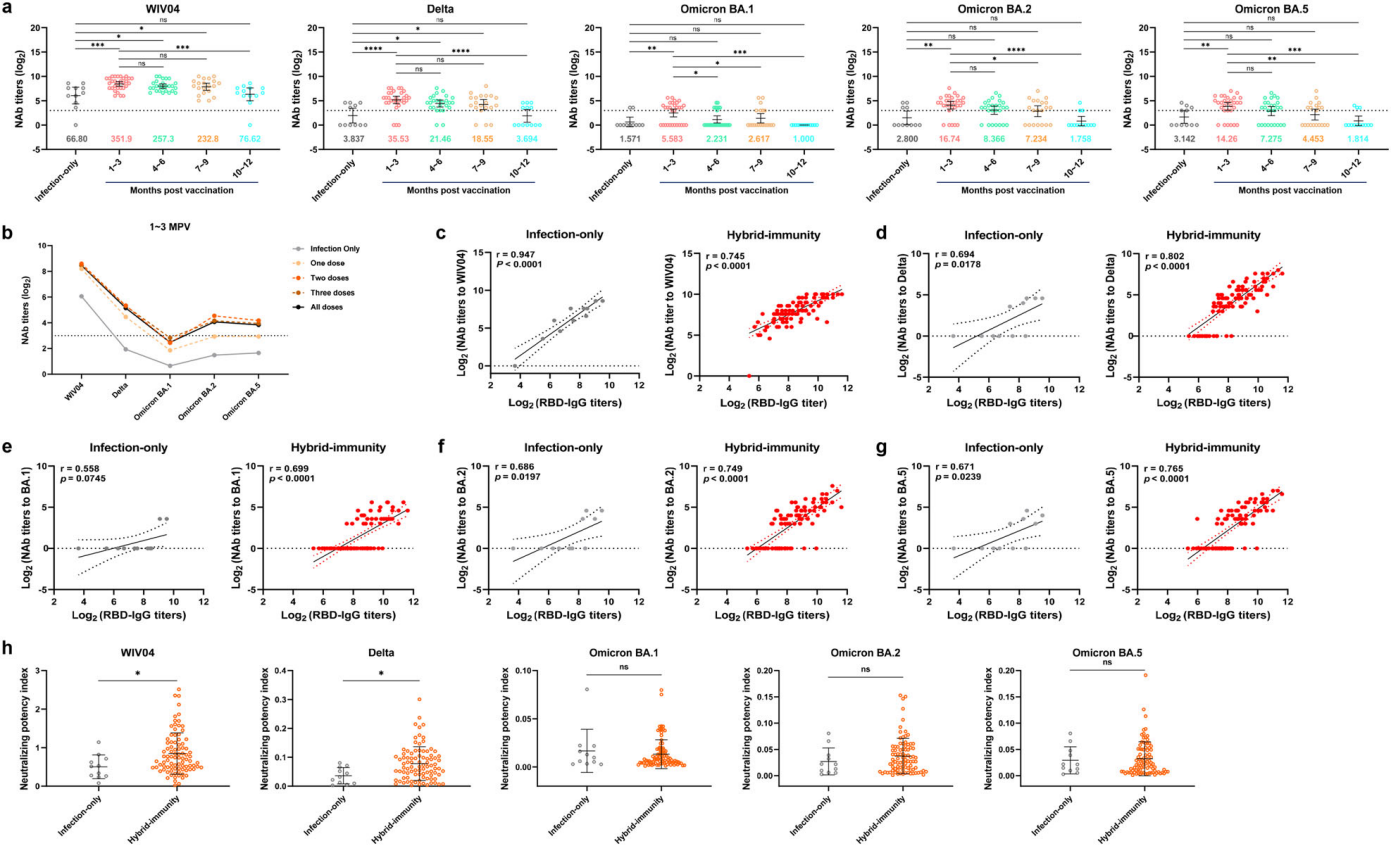
Neutralization efficiency of serum antibodies against authentic SARS-CoV-2. **a** NAb titers against the authentic SARS-CoV-2 WIV04, Delta, and Omicron BA.1 and BA.2 strains in the hybrid-immunity group at 1–3, 4–6, 7–9, and 10–12 months post vaccination and in the infection-only group. The dotted line represents the limit of detection (1:8); below the limit of detection, values were set to 1:1. Error bars represent the GMT values with 95% confidence intervals, and the colored numbers in the figures represent GMT values. Statistical significance was analyzed using the Kruskal–Wallis test. **b** GMT values against the authentic SARS-CoV-2 strains in the infection-only group and hybrid-immunity group (one, two, three, and all doses, at 1–3 months post vaccination). **c**–**g** Correlation between NAb titers (GMT) against WIV04 (**c**), Delta (**d**), Omicron BA.1 (**e**), BA.2 (**f**), and BA.5 (**g**) and RBD-IgG titers in the infection-only (left) and hybrid-immunity (right) groups. Scatter plots represent a simple linear fit with 95% confidence bands, together with the Spearman correlation coefficient and 2-tailed *P* value. **h** The ratio of authentic SARS-CoV-2 NAb titers (IC_50_) to RBD-IgG titers (dilution quantitative) was calculated as a neutralization potency index. Data are means ± SD. Statistical significance was analyzed using the paired *t-*test. WIV04, SARS-CoV-2 nCoV-2019BetaCoV/Wuhan/WIV04/2019; MPV, months post vaccination.

The GMT values of NAb titers based on the authentic SARS-CoV-2 were significantly elevated in the hybrid-immunity group compared with those in the infection-only group for WIV04 and Delta up to 7–9 months post vaccination but persisted for only 1–3 months post vaccination for Omicron (Fig. 3a). Consistent with the results for pseudotyped SARS-CoV-2, regardless of the number of vaccine doses the patients received, the GMT values of NAb titers for all tested strains showed a time-dependent decline in the hybrid-immunity group (Supplementary Fig. S4). The NAb GMT results showed that Delta and Omicron BA.1, BA.2, and BA.5 were all highly resistant to neutralization by infection-only group serum samples, with only 45.5% (5/11) for Delta, 18.2% (2/11) for Omicron BA.1, 36.4% (4/11) for Omicron BA.2, and 45.5% (5/11) for Omicron BA.5 exhibiting NAb titers above the detection limit (Fig. 3a, b). The NAb GMT values of serum samples from patients in the hybrid-immunity group within 3 months post vaccination were 5.3-, 9.3-, 3.6-, 6.0- and 4.5-fold higher than those from the infection-only group for the WIV04, Delta, Omicron BA.1, BA.2 and BA.5 strains, respectively. The positive rates for these five SARS-CoV-2 strains were 100%, 90%, 60%, 83.3%, and 80%, respectively (Fig. 3a, b). However, compared with the effects against the WIV04 or Delta strain, the serum samples from the hybrid-immunity group showed weak NAb titers against Omicron BA.1, BA.2, and BA.5 within 3 months post vaccination (Fig. 3a). Furthermore, the neutralizing activity against both the WIV04 and Delta strains remained at high levels for 6–9 months post vaccination. However, the NAb titers against Omicron declined more rapidly, and the high level persisted for only 4–6 months or even 1–3 months post vaccination (Fig. 3a).

Similar to the pseudotyped virus experimental results, the correlation between the IC_50_ titers of NAbs to authentic SARS-CoV-2 and titers of RBD-IgG showed that the quality and breadth of humoral immunity could be improved after inoculation with the inactivated vaccines. For all five authentic SARS-CoV-2 strains, neutralizing titers were related to RBD-IgG levels for both the infection-only and hybrid-immunity groups, but the hybrid-immunity group had a smaller *P* value (Fig. 3c–g). Additionally, RBD-IgG levels were correlated more strongly with NAb titers against Delta and Omicron BA.1, BA.2, and BA.5 strains in the hybrid-immunity group (Fig. 3c–g). To further analyze the neutralization efficiency of sera against a given SARS-CoV-2 strain, the ratio of the NAb titer (IC_50_) to RBD-IgG titer (dilution quantitative) was calculated (hereafter referred to as the neutralizing potency index)^24,32^. The results showed that the neutralizing potency indexes for the WIV04 and Delta strains were relatively higher in the hybrid-immunity group than in the infection-only group (Fig. 3h). These findings further support the beneficial outcomes of humoral immunity to SARS-CoV-2 that can be achieved through vaccination of recovered patients. Last, we found that the neutralization efficiency of serum antibodies for pseudotyped and authentic SARS-CoV-2 was positively correlated (Supplementary Fig. S5).

### Determination of specific CD4^+^ T cell responses to SARS-CoV-2

**Fig. 4:**
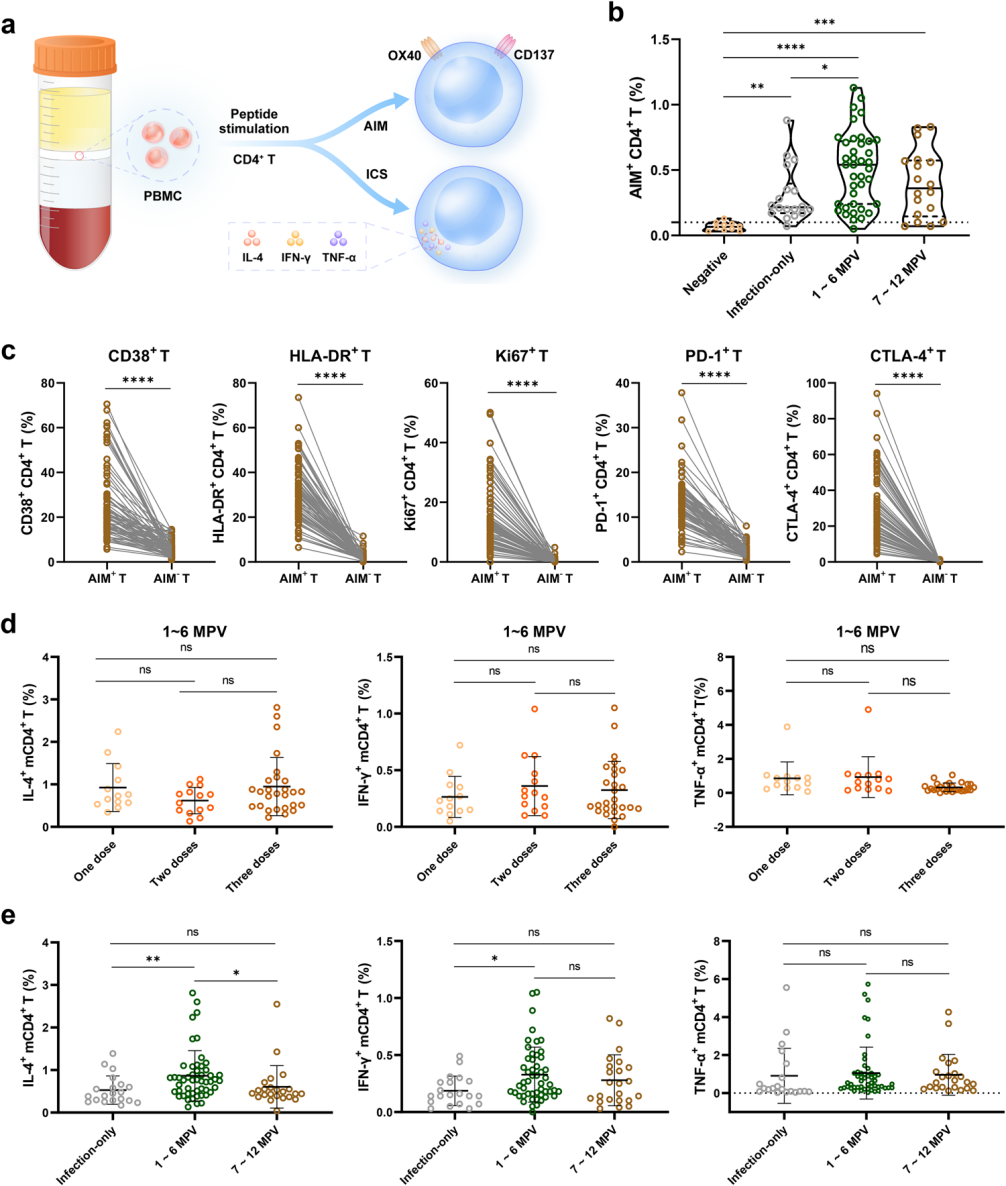
SARS-CoV-2-specific CD4^+^ T cell response. **a** Schematic diagram depicting the CD4^+^ T-cell AIM assay. **b** The percentage of AIM^+^ (CD137^+^ and OX40^+^) CD4^+^ T cells was detected after stimulation with the SARS-CoV-2 peptide pool for 24 h. Ten samples in the hybrid-immunity group without stimulation from the peptide pool were chosen as negative controls. Seventy-five recovered patients in the hybrid-immunity group with different RBD-IgG titers and immunization schedules were selected for CD4^+^ T-cell analysis. Each dot represents an individual. Lines denote the median and quartile. **c** Pairwise comparison of the expression of the surface markers CD38, HLA-DR, PD-1 and CTLA-4 and the proliferation marker Ki67 in AIM^+^ CD4^+^ T cells or AIM^–^ (OX40^–^CD137^–^) CD4^+^ T cells. Statistical significance was determined using the paired *t*-test. **d** The percentage of SARS-CoV-2-specific mCD4^+^ T cells (secreting IL-4, TNF-α or IFN-γ) detected after stimulation by the SARS-CoV-2 peptide pool in the hybrid-immunity group individuals with one, two or three doses of vaccination at 1–6 months post vaccination. **e** Comparison of the percentages of different mCD4^+^ cell subsets (IL-4^+^, IFN-γ^+^, and TNF-α^+^) from the infection-only and hybrid-immunity groups at 1–6 months or 7–12 months post vaccination. Statistical significance was analyzed using the Kruskal–Wallis test in **b**, **d**, and **e**. Only groups with significant differences are marked in **b**. MPV, months post vaccination; AIM, activation-induced marker; ICS, intracellular cytokine staining.

A T cell receptor (TCR)-dependent AIM assay was employed to determine SARS-CoV-2-specific CD4^+^ T cell responses in recovered COVID-19 patients immunized with COVID-19 vaccines, which has been successfully used to reveal vaccine-specific or virus-specific CD4^+^ T cells in several studies^33,34^. SARS-CoV-2-specific CD4^+^ T cells were evaluated by incubating PBMCs with a SARS-CoV-2 prototype peptide pool for stimulation (Fig. 4a). Following incubation, SARS-CoV-2-specific CD4^+^ T cells were identified using activation markers OX40 and CD137 (Supplementary Fig. S6). Compared with that in the infection-only and hybrid-immunity groups (7–12 months post vaccination), the proportion of SARS-CoV-2-specific CD4^+^ T cells in the hybrid-immunity group within 6 months post vaccination was 1.65-fold (*P* < 0.05) and 1.28-fold higher, respectively (Fig. 4b). The expression of proliferation marker Ki67 in AIM^+^ CD4^+^ T cells was dramatically upregulated in the individuals within 1–6 months post vaccination compared to the expression in those within 7–12 months post vaccination or in the infection-only group (Supplementary Fig. S7a). Although not dramatic, the expression of the exhaustion marker CTLA-4 on AIM^+^ CD4^+^ T cells in the infection-only group was elevated by 1.29-fold compared with that in the hybrid-immunity group within 1–6 months post vaccination (Supplementary Fig. S7a). Analysis of the expression of HLA-DR, CD38 and PD-1 on AIM^+^ CD4^+^ T cells indicated no difference between the infection-only and hybrid-immunity groups (Supplementary Fig. S7a). Moreover, AIM^+^ CD4^+^ T cells expressed remarkably higher levels of activation markers (CD38 and HLA-DR), proliferation marker (Ki67) and exhaustion markers (CTLA-4 and PD-1) than AIM^–^ CD4^+^ T cells (Fig. 4c). In total, the SARS-CoV-2-specific CD4^+^ T immune response was stronger within 6 months and the intensity decreased over time after vaccination. However, the remaining SARS-CoV-2-specific CD4^+^ T cells could be reactivated for a rapid response upon viral re-exposure.

To provide novel insight into the functionality of long-term SARS-CoV-2-specific memory CD4^+^ T (mCD4^+^ T) cells in recovered COVID-19 patients with or without vaccination, SARS-CoV-2-specific mCD4^+^ T (CCR7^–^, CD45RA^–^) cells positive for the intracellular cytokines TNF-α, IFN-γ and IL-4 were examined by a multiparameter ex vivo ICS assay. The negative control (samples without stimulation by the peptide pool) exhibited only low proportions of TNF-α^+^, IFN-γ^+^ and IL-4^+^ mCD4^+^ T cells (Supplementary Fig. S7b). The 16 samples stimulated with phorbol myristate acetate (PMA), as a positive control, presented higher percentages of TNF-α^+^, IFN-γ^+^, IL-4^+^, or IL-2^+^ mCD4^+^ T cells, which showed no correlation with the RBD-IgG titers (Supplementary Fig. S7c).

There were no significant differences in the percentages of any of these three types of mCD4^+^ T cells (IL-4^+^, IFN-γ^+^, or TNF-α^+^) within 6 months post vaccination among the recovered individuals who received one, two, or three doses of inactivated SARS-CoV-2 vaccines (Fig. 4d). The proportion of these mCD4^+^ T cells was comparable between 1–3 months and 4–6 months post vaccination (Supplementary Fig. S7d), which was consistent with the RBD-IgG titers (Fig. 1d). The percentages of IL-4^+^ and IFN-γ^+^ mCD4^+^ T cells in the hybrid-immunity group within 6 months post vaccination were significantly higher than those in the infection-only group, and the percentage of TNF-α^+^ mCD4^+^ T cells in the hybrid-immunity group was comparable to that in the infection-only group (Fig. 4e). Additionally, positive TNF-α^+^, IFN-γ^+^, and IL-4^+^ mCD4^+^ T cells could still be detected in the infection-only group (Fig. 4e).

### Functional correlation of mCD4^+^ T cell responses

**Fig. 5:**
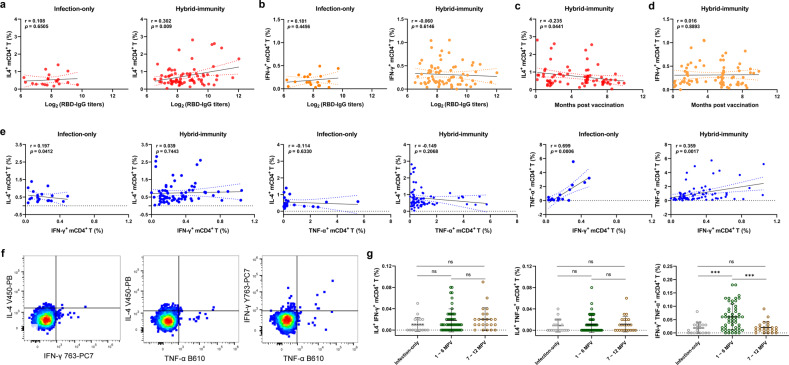
The function of SARS-CoV-2-specific mCD4^+^ T-cell responses in the infection-only and hybrid-immunity groups upon SARS-CoV-2-specific peptide pool stimulation. **a**, **b** Correlation between the percentage of IL-4^+^ (**a**) or IFN-γ^+^ mCD4^+^ T cells (**b**) and the magnitude of RBD-IgG titers in the infection-only group and hybrid-immunity group. **c**, **d** Correlation between the percentage of IL-4^+^ (**c**) or IFN-γ^+^ (**d**) mCD4^+^ T cells and the number of months post vaccination in the hybrid-immunity group. **e** Correlation between the percentages of any of two types of IL-4^+^, IFN-γ^+^ or TNF-α^+^ mCD4^+^ T cells in the infection-only group and hybrid-immunity group. **f** The multiple functions (cytokine production) of SARS-CoV-2-specific mCD4^+^ T cells. **g** Comparison of the percentages of different multifunctional mCD4^+^ cell subsets (IL-4^+^IFN-γ^+^, IL-4^+^TNF-α^+^, and IFN-γ^+^TNF-α^+^) from the infection-only and hybrid-immunity groups at 1–6 months or 7–12 months post vaccination. Statistical significance was analyzed using the Kruskal–Wallis test. Scatter plots in **a**–**e** depict the simple linear fit with 95% confidence bands, together with the Spearman correlation coefficient and 2-tailed *P* value. MPV, months post vaccination.

CD4^+^ T cells are involved in the majority of protective antibody responses, and the IL-4^+^ mCD4^+^ T cell frequencies were correlated well with the RBD-IgG and NP-IgG titers in the hybrid-immunity group, but such a correlation was not observed in the infection-only group (Fig. 5a; Supplementary Fig. S7e). A negative correlation was found between the frequency of IL-4^+^ mCD4^+^ T cells and number of months post vaccination (Fig. 5c). However, no correlation was found for IFN-γ^+^ mCD4^+^ T cells (Fig. 5b, d).

Next, the positive correlation between SARS-CoV-2-specific TNF-α^+^ and IFN-γ^+^ mCD4^+^ T cells was confirmed, but the frequency of SARS-CoV-2-specific IL-4^+^ mCD4^+^ T cells was not well correlated with that of TNF-α^+^ or IFN-γ^+^ mCD4^+^ T cell responses (Fig. 5e). Regarding the multiple functions of SARS-CoV-2-specific mCD4^+^ T cells, the percentage of multifunctional mCD4^+^ T cells (two of IL-4^+^, IFN-γ^+^, and TNF-α^+^) was lower than that of single cytokine-producing mCD4^+^ T cells (Fig. 5f). There was no increase in the proportion of multifunctional IL-4^+^IFN-γ^+^ or IL-4^+^TNF-α^+^ mCD4^+^ T cells found in the hybrid-immunity group, while the proportion of multifunctional IFN-γ^+^TNF-α^+^ mCD4^+^ T cells increased significantly within 6 months post vaccination (Fig. 5g).

## Discussion

It has been more than two years since the emergence of the COVID-19 pandemic in December 2019. However, recovered patients had only limited levels of SARS-CoV-2-specific antibodies two years after recovery from COVID-19 (Fig. 1d), and neutralizing activity against the new mutant strains was substantially weakened in most serum samples (Fig. 3a), indicating that natural infection alone does not provide long-term protection against reinfection. As of September 10, 2022, SARS-CoV-2 has infected more than 607 million people worldwide^1^, and many of them suffer from severe and long-term sequelae^35,36^. COVID-19 remains the greatest threat to global public health. Therefore, we should pay more attention to the significance of vaccination for preventing SARS-CoV-2 infection.

Accumulating evidence has shown that the production of SARS-CoV-2-specific antibodies (RBD-IgG) decreases over time in individuals who recovered from COVID-19, where only 35.7% of the initial level persisted after 9 months, and the GMT range for RBD-IgG titers was 156–189 thereafter until 1 year after recovery, in our previous study^7^. In comparison, the RBD-IgG titer was 143 in the infection-only group 23–25 months post recovery (Fig. 1d). The results indicated that the antibody titers of infection-only individuals remained stable for 1–2 years post recovery, therefore it is reasonable to consider that the infection-only group could be the background population to reflect the immune features activated by vaccination in recovered individuals. The recovered patients receiving an inactivated vaccine had greater potency, quality, and breadth of response to neutralize SARS-CoV-2 compared with that of recovered patients without vaccination (the infection-only group), which was consistent with the findings of mRNA vaccine studies^24,30,37^. The authentic virus GMT values (neutralization potency) of the hybrid-immunity group against the prototype, Delta, and Omicron BA.1, BA.2, and BA.5 strains were 6.0- to 9.3-fold higher than those of the infection-only group. The positive rates for these five SARS-CoV-2 strains were 100%, 90%, 60%, 83.3%, and 80% in the hybrid-immunity group and 90.9%, 45.5%, 18.2%, 36.4%, and 45.5% in the infection-only group, respectively (Fig. 3a, b). Vaccination of recovered patients was associated with a significantly increased proportion of SARS-CoV-2-specific NAbs (Fig. 3c–h), and the improvement in NAb quality and breadth could be caused by the increased levels of memory B cells^25,38^.

However, there are only a few long-term studies on sustainable neutralization activity in serum after immunization^39^. Our results showed that RBD-IgG titers, NP-IgG titers, and serum neutralization potency could all be maintained for 4–6 months post vaccination, although there was an obvious decline 7–9 months post vaccination (Figs. 1–3). Hence, the reinduced durability of NAbs in recovered patients is comparable to that in vaccinated individuals who have never been infected^40,41^. In addition, the maintenance time of neutralization for mutant strains began to decline, especially for Omicron; the neutralizing activity persisted for only 1–3 months post vaccination, and the neutralization potency for Omicron BA.1, BA.2, and BA.5 relative to that for the prototype was decreased by 63.0-fold, 21.0-fold and 24.7-fold, respectively (Fig. 3). With vaccination after SARS-CoV-2 prototype infection, the neutralizing activity of serum antibodies against Omicron was lower than that against the prototype or Delta strain. The reduced neutralization activity might be due to the large number of mutations in the S protein of Omicron subvariants, including extensive changes in both the RBD and NTD^37,42^. Accumulating mutations in the S protein have been predicted to affect NAb epitopes and reduce antibody neutralization activity (antigenic drift)^43,44^. In the context of the Omicron pandemic, a vaccine developed based on the prototype strain will still need to be administered every 6 or even 3 months to reach comparable protection efficiency. Such a vaccination frequency is not advisable, and thus, the development of vaccines against Omicron is extremely crucial. Last, due to the emergence of different SARS-CoV-2 variants, the demand for the Omicron vaccine depends on the staying power of the variants; thus, bivalent or polyvalent vaccines may be good candidates to prevent immune escape.

In addition, our results showed that the RBD-IgG titers of recovered patients rose rapidly within 3 months post vaccination (Fig. 1c, d) but did not exceed or reach the initial level (the 1st and 2nd months following diagnosis)^7^. This finding also explains why the duration of SARS-CoV-2-specific IgG after vaccination cannot be longer than that of natural immunity. After natural infection, some studies have shown that severe clinical manifestations lead to high titers of anti-SARS-CoV-2 antibodies^45,46^, and antibody titers are also affected by sex (with higher titers in men)^7,47^. In contrast to these reports, we found that these associations were no longer detectable 2 years after recovery, regardless of prior vaccination. The relationship between antibody titers and age was a negative correlation in children and a positive correlation in adults after natural infection^48^, but there was no significant correlation between antibody titers and age in adults aged 30–50 years observed in this study (Fig. 1g). We speculate that the disappearance of such correlations may be due to immunity returning to normal and the fact that individuals with a strong immune response after natural infection do not maintain high-strength immune memory against SARS-CoV-2 over a long time period.

Apart from antibodies, viral-specific T cells contribute to viral clearance during acute infection. The humoral immune responses were decreased several months after recovery, suggesting that SARS-CoV-2 infection induces a strong response in the short term, but that response is gradually lost and does not provide enough long-term protection^8^. Consistently, we observed that the frequency of AIM^+^ CD4^+^ T (SARS-CoV-2-specific CD4^+^ T) cells in the infection-only group was much lower than that in the hybrid-immunity group (Fig. 4b). The main reason was speculated to be that the immune response was enhanced by vaccination. It was found that the expression levels of immune checkpoint markers (PD-1, CD38, HLA-DR, and CTLA-4) were all markedly increased on AIM^+^ CD4^+^ T cells compared to those on non-SARS-CoV-2-specific AIM^–^ CD4^+^ T cells (Fig. 4c), which aligned with the findings of early investigations^11,49^. In contrast, the comparable expression levels of some immune checkpoint markers between the infection-only group and the hybrid-immunity group (Supplementary Fig. S7a) indicated fewer signs of functional alterations in the activated cells.

Previous studies demonstrated that there was no significant reduction in the SARS-CoV-2-specific CD4^+^ T cell population in recovered patients 12 months post infection compared with that 6 months after disease onset^50^. However, the intensity of the IL-4^+^ mCD4^+^ T cell response at 7–12 months post vaccination in the hybrid-immunity group was not different from that in the infection-only group, while it was lower than that at 1–6 months post vaccination (Fig. 4e). This result suggests that the persistence of the IL-4^+^ mCD4^+^ T cell response induced by the inactivated SARS-CoV-2 vaccine in recovered patients does not exceed that with natural infection^51^. A significant correlation between the percentage of SARS-CoV-2-specific IL-4^+^ mCD4^+^ T cells (regulating acquired antibody responses) and the magnitude of RBD-IgG titers within 12 months post vaccination was found in the COVID-19 convalescent patients with vaccination (Fig. 5a), supporting the association between antibody responses and CD4^+^ T cells in some long-term cohorts^4,9,52^. Moreover, a negative correlation between the SARS-CoV-2-specific IL-4^+^ mCD4^+^ T cell response and the length of vaccination was also observed (Fig. 5c), indicating a decreased intensity of the SARS-CoV-2-specific T helper 2 cell response that could influence antibody production over time.

In this cohort, based on the correlation analysis, the response of SARS-CoV-2-specific IFN-γ^+^ mCD4^+^ T cells was not correlated with the magnitude of the RBD-IgG titer or IL-4^+^ mCD4^+^ T response (Fig. 5b, e), indicating that neither RBD-IgG nor functional humoral immunity is a good proxy to estimate the cellular response to SARS-CoV-2. Moreover, the percentage of IFN-γ^+^ mCD4^+^ T cells in the hybrid-immunity group within six months was significantly higher than that in the infected-only group (Fig. 4e). The insufficient magnitude or durability of memory cellular and humoral immunity in the infection-only group would result in failure to maintain efficient immunity, and type-I interferon immunity is important for early protective immunity against COVID-19^53,54^.

Additionally, the IFN-γ^+^ mCD4^+^ T cell response was positively correlated with the TNF-α^+^ mCD4^+^ T cell response (Fig. 5e), indicating that these two subpopulations of mCD4^+^ T cells work in concert in the specific cellular immune response to SARS-CoV-2^55,56^. Previous studies demonstrated that SARS-CoV-2-specific CD4^+^ T cells exhibited multifunctionality (expressing both IFN-γ and TNF-α) in patients who recovered within 3–8 weeks^23^, and multifunctional T cells secreting TNF-α and IFN-γ could also be detected in individuals receiving SARS-CoV-2 mRNA, inactivated and adenovirus-vectored vaccines^51,57,58^. In our study, the percentage of IFN-γ^+^TNF-α^+^ mCD4^+^ T cells significantly decreased at 7–12 months compared with that at 1–6 months post vaccination and returned to levels consistent with those of the infection-only group 7–12 months post vaccination (Fig. 5g). Therefore, the magnitude of the multifunctional T cell response gradually decreased in COVID-19 patients after two years of recovery, and the IFN-γ^+^TNF-α^+^ multifunctional SARS-CoV-2-specific mCD4^+^ T cell response could be activated post SARS-CoV-2 vaccination. For CD4^+^ T cells, 95% of the epitopes spanning the entire SARS-CoV-2 proteome are fully conserved across variants, and thus vaccinated but SARS-CoV-2-naïve individuals also produce CD4^+^ T cell responses following stimulation with the SARS-CoV-2 variant peptide pool^59^. Due to this property of T cell epitopes, SARS-CoV-2-specific CD4^+^ T cell reactivity can be evaluated by using the prototype peptide pool, and thus, the SARS-CoV-2 variant peptide pool was not used in this study.

In conclusion, the present study demonstrated that recovered COVID-19 patients receiving either one dose or multiple doses of the vaccine all have significantly larger booster effects on the potency and breadth of NAbs and the magnitude of humoral and cellular immune responses, which would maintain neutralizing activity against both the prototype and SARS-CoV-2 mutants over a longer period. To achieve global vaccination, individuals with previous COVID-19 should not be neglected, and booster vaccination should be performed every 6–9 months as SARS-CoV-2 is endemic, especially the mutant strains. More importantly, vaccines against Omicron should be developed to address its widespread and immune-evasive nature.

## Materials and methods

### Recovered COVID-19 patients and specimen collection

The study was performed in accordance with the Declaration of Helsinki and reviewed for approval by the Ethics Committee of Tiantan Biological R&D Center (approval No. TTSW-EC2021-05). All participants were over 18 years old, and written informed consent was obtained at the time of their enrollment. All participants had no breakthrough infection or reinfection during the period between primary infection and blood collection.

Serum (10 mL of whole blood) or PBMCs (15 mL of whole blood) were collected into a blood collection tube (Chengwu Medical, China) containing procoagulant or heparin sodium. Serum samples were obtained immediately after centrifugation at 1500 rpm for 15 min and then stored at −20 °C for serology assays. PBMCs were obtained via density gradient centrifugation using Ficoll-Paque (Lymphoprep, STEMCELL Technologies) according to the guidance for use. The isolated PBMCs were resuspended in cryopreserved cell recovery medium containing 10% dimethyl sulfoxide (DMSO; Gibco) and 90% heat-inactivated fetal bovine serum (Gibco) and then stored in liquid nitrogen until cellular assays were conducted.

### ELISA

ELISA was conducted as described previously^7^. The RBD-IgG and NP-IgG titers for all serum samples were detected using commercial SARS-CoV-2 IgG ELISA (qualitative) kits (Beijing WanTai Biological Pharmacy Enterprise Co., Ltd., China). These two kits employed an indirect, solid phase ELISA method for detection. The quantitative detection method was established by using internal control references. The target value was presented as the dilution value, and the highest dilution value was indicated by an optical density (OD) value greater than the cutoff value. COVID-19 post vaccination pooled plasma was used as the internal control reference for RBD-IgG and NP-IgG, and its dilution value was 1:320 (Lot No.: ZB2021042003, Sinopharm Wuhan Plasma-derived Biotherapies Co., Ltd.). They were diluted with the sample buffer in the kit via 2-fold serial dilution (1:10, 1:20, 1:40, 1:80, 1:160, 1:320, 1:640, and 1:1280), and a 100 μL aliquot of diluted reference samples was added into the standard well. Then, 10 μL of samples for detection were added to the sample wells containing 100 μL of sample buffer, conforming to the kit instructions. The OD in each well was detected by a SpectraMAX plus384 at 450 nm (Molecular Devices, CA, USA), and the data were processed with SoftMax5.2 software. A standard curve was established by fitting to a four-parameter equation, using the OD value as the ordinate and the dilution value as the abscissa. The recovery rate of each standard dilution ≤ 20% of the target value and R^2^ value > 0.99 were considered the acceptable criteria for the linearity of the standard curve. The present detection method has a detection range of dilution values from 10 to 320. The corresponding dilution value was calculated by the standard curve based on the OD value of each sample. To obtain accurate results, the samples that exceeded the upper limit of detection were further diluted.

### Pseudotyped virus-based SARS-CoV-2-neutralizing antibody assay

Pseudotyped virus-based neutralization assays were performed as previously described^60^. The NAb activity against the pseudotyped virus was determined by the reduction in luciferase gene expression. Wuhan-1 (GenBank: MN908947), Delta, Omicron BA.1, and omicron BA.2 pseudotyped viruses were obtained from Gobond Science and Technology (Beijing) Co., Ltd. (China). Serum samples were inactivated for 30 min at 56 °C and sterile-filtered via a syringe filter (0.22 µm). The inactivated serum was serially diluted by 4-fold with DMEM (Gibco) in a 96-well cell plate and preincubated with equal volumes of pseudotyped viruses (1–3 × 10^4^ TCID_50_ mL^–1^). Subsequently, 2 × 10^4^ Huh-7 cells (TCHu182; National Collection of Authenticated Cell Cultures, Shanghai, China) were added to each well and incubated in 5% CO_2_ for 20–24 h at 37 °C. Pseudotyped virus + Huh-7 cells and cells only were used as the virus control and background control, respectively. To leave 100 µL in each well, the culture supernatant was gently aspirated. Then, 100 µL of luciferase substrate (PerkinElmer, MA, USA) was added to each well and incubated for 2 min at room temperature. After that, 150 µL of lysate was transferred to 96-well white solid plates, and the luminescence signals were detected using a microplate luminometer (PerkinElmer, MA, USA). The positive well was defined as relative luminescence unit values 10-fold greater than the cell background. The IC_50_ values were calculated using the Reed–Muench method^61^. IC_50,_ the highest serum dilution ratio with a 50% neutralization rate, was employed to assess NAb potency, and a dilution titer over 2^5^ was considered positive. Mutation information for pseudotyped viruses expressing the SARS-CoV-2 S protein is presented in Supplementary Table S2.

### Neutralizing antibody tests based on the authentic SARS-CoV-2 microneutralization assay

Vero cells (TL-CCL-81.4; American Type Culture Collection, VA, USA) were grown in 96-well plates and incubated overnight. Serum specimens were serially diluted by 2-fold starting from the 1:8 dilution. Then, the cells were incubated with an equivalent amount of 100 TCID_50_ of SARS-CoV-2 for 2 h at 37 °C. Subsequently, the Vero cell suspension (1.5–2.0 × 10^5^ cells/mL) was added to the serum–virus mixture and then incubated for 3–5 days at 37 °C. Both virus-only and cell-only wells were used as controls. The NAb titer was calculated as the reciprocal dilution of serum neutralizing 50% of virus infection of the cells. The authentic virus neutralization assays were conducted in a biosafety level-3 laboratory. SARS-CoV-2 authentic viruses of the WIV04 (NCBI Reference Sequence: NC_045512.2), Delta (GenBank accession No. OU428631.1), Omicron BA.1 (GISAID accession No. EPI_ISL_7138045), Omicron BA.2 (GISAID accession No. EPI_ISL_13867424), and Omicron BA.5 (GISAID accession No. EPI_ISL_11873915) strains were used in the assays.

### Synthetic peptides

The peptide pool used in this study contains 42 peptides from SARS-CoV-2 virus S, M, NP, ORF-3a, and ORF-7a proteins, as described previously (Supplementary Table S3)^11,62^. All the peptides were synthesized and lyophilized as crude material (purity: > 95%, GenScript, China). All of these individual peptide aliquots (4 mg/aliquot) were pooled into a mega pool and then subjected to lyophilization and resuspension in DMSO at 1 mg/mL each.

### AIM assay

The cryopreserved PBMCs were thawed in a water bath at 37 °C. Then, the cells were rinsed twice and cultured with complete RPMI 1640 (Gibco) with 10% FBS. After 5 h of resting, PBMCs were incubated for 19 h with the SARS-CoV-2 peptide pool (2 μg/mL) in a 12-well cell culture plate at 1 × 10^6^ PBMCs/well. A negative control without peptide stimulation was included for 10 individuals. For the AIM assay, the cells were stained with Live/Dead Fixable Viability Stain 440U (BD). Antigen-specific CD4^+^ T cells were collected and counted to obtain the percentage of (CD3^+^CD4^+^OX40^+^CD137^+^) T cells, while CD38, Ki67, CTLA-4, PD-1 and HLA-DR (BioLegend) were tested as AIM markers. All the antibodies employed in this study can be found in Supplementary Table S4.

### ICS assay

The PBMCs were rested for 5 h and stimulated for 19 h at 37 °C in 5% CO_2_ with the SARS-CoV-2 peptide pool (2 μg/mL), followed by an additional 5 h of incubation with 5 μg/mL brefeldin A (BioLegend). An equal volume of DMSO was used as a negative control (*n* = 10), while an activation cocktail with brefeldin A (BioLegend)-stimulated cells was employed as a positive control (*n* = 16). After culture, the cells were stained with Live/Dead marker (BD) for 15 min in the dark and rinsed with PBS. Antibodies against the surface markers CD3, CD4, CD45RA, and CCR7 and the intracellular cytokine markers IFN-γ, TNF-α, and IL-4 were directly added to the cells and incubated at 4 °C for 60 min in the dark. Then, the cells were rinsed twice with Intracellular Staining Perm Wash Buffer (BioLegend) and fixed with Fixation Buffer (BioLegend) overnight at 4 °C. All the antibodies utilized for flow cytometry can be found in Supplementary Table S4.

All specimens were analyzed using CytoFLEX S Flow Cytometry (Beckman Coulters), and the data were processed with CytoExpert software (Beckman Coulters). A representative gating strategy for AIM- or cytokine-positive cells was based on the negative controls, known as unstimulated samples. Briefly, the lymphocytes were gated, and the counts of single cells were determined. All T cell data were gated for negative to Live/Dead dye staining and positive to CD3^+^ staining. A representative gating strategy for the AIM and ICS assays is illustrated in Supplementary Fig. S5. The sample with a weak live CD3^+^ signal was excluded as a quality control.

### Statistical analysis

The antibody titers (RBD-IgG, NP-IgG, and NAb against pseudotyped and authentic viruses) are presented as GMT values with 95% confidence intervals. The IC_50_ value of pseudotyped virus-based neutralization activity below the limit of detection (1:5) was set to 1:1. The IC_50_ value of the authentic virus microneutralization test below the limit of detection (1:8) was set to 1:1. The neutralizing potency index and flow cytometry results are presented as the mean with standard deviation (SD). For more than two groups, data normality was confirmed with Anderson‒Daring, Shapiro‒Wilk and Kolmogorov‒Smirnov tests. The Kruskal–Wallis test was selected according to the normal distribution and homogeneity of variance. The Wilcoxon matched-pairs signed rank test was employed for the paired *t-*test of two groups. The Spearman rank correlation test for nonparametric data was applied to determine the association between 2 factors. Statistical significance was analyzed (*P < 0.05, **P < 0.01, ***P < 0.001, ****P < 0.0001). All statistical tests were conducted using GraphPad Prism 9.0 (CA, USA).

## Supplementary information

Supplementary information

Supplementary Data S1

Supplementary Data S2

Supplementary Data S3

Supplementary Data S4

Supplementary Data S5

Supplementary Data S6

Supplementary Data S7

Supplementary Data S8

Supplementary Data S9

Supplementary Data S10

Supplementary Data S11

## Data Availability

Data supporting the findings of this study are available within the paper or its supplementary materials. Source data for Figs. 1–5 and Supplementary Figs. S1–S5, S7 can be found in Supplementary Data S1–S11. Other data that support the findings of this study are available from the corresponding author upon reasonable request.

## References

[1] Dong E, Du H, Gardner L (2020). An interactive web-based dashboard to track COVID-19 in real time. Lancet Infect. Dis..

[2] Ai J (2022). Antibody evasion of SARS-CoV-2 Omicron BA.1, BA.1.1, BA.2, and BA.3 sub-lineages. Cell Host Microbe.

[3] Pulliam JRC (2021). Increased risk of SARS-CoV-2 reinfection associated with emergence of the Omicron variant in South Africa. Science.

[4] Dan JM (2021). Immunological memory to SARS-CoV-2 assessed for up to 8 months after infection. Science.

[5] Wajnberg A (2020). Robust neutralizing antibodies to SARS-CoV-2 infection persist for months. Science.

[6] He Z (2021). Seroprevalence and humoral immune durability of anti-SARS-CoV-2 antibodies in Wuhan, China: a longitudinal, population-level, cross-sectional study. Lancet.

[7] Li C (2021). Twelve-month specific IgG response to SARS-CoV-2 receptor-binding domain among COVID-19 convalescent plasma donors in Wuhan. Nat. Commun..

[8] Bonifacius A (2021). COVID-19 immune signatures reveal stable antiviral T cell function despite declining humoral responses. Immunity.

[9] Zuo J (2021). Robust SARS-CoV-2-specific T cell immunity is maintained at 6 months following primary infection. Nat. Immunol..

[10] Lu Z (2021). Durability of SARS-CoV-2-specific T-cell responses at 12 months postinfection. J. Infect. Dis..

[11] Hou H (2021). Immunologic memory to SARS-CoV-2 in convalescent COVID-19 patients at 1 year postinfection. J. Allergy Clin. Immunol..

[12] Xia S (2021). Safety and immunogenicity of an inactivated COVID-19 vaccine, BBIBP-CorV, in people younger than 18 years: a randomised, double-blind, controlled, phase 1/2 trial. Lancet Infect. Dis..

[13] Xia S (2020). Effect of an inactivated vaccine against SARS-CoV-2 on safety and immunogenicity outcomes: interim analysis of 2 randomized clinical trials. JAMA.

[14] Huang B (2021). Serum sample neutralisation of BBIBP-CorV and ZF2001 vaccines to SARS-CoV-2 501Y.V2. Lancet Microbe.

[15] Baden LR (2020). Efficacy and safety of the mRNA-1273 SARS-CoV-2 vaccine. N. Engl. J. Med..

[16] Wang H (2020). Development of an inactivated vaccine candidate, BBIBP-CorV, with potent protection against SARS-CoV-2. Cell.

[17] Sadoff J (2021). Safety and efficacy of single-dose Ad26.COV2.S vaccine against Covid-19. N. Engl. J. Med..

[18] Polack FP (2020). Safety and efficacy of the BNT162b2 mRNA Covid-19 vaccine. N. Engl. J. Med..

[19] Shang J (2020). Cell entry mechanisms of SARS-CoV-2. Proc. Natl. Acad. Sci. USA.

[20] Chi X (2020). A neutralizing human antibody binds to the N-terminal domain of the Spike protein of SARS-CoV-2. Science.

[21] Sanders, B., Koldijk, M., Schuitemaker, H. In *Vaccine Analysis: Strategies, Principles, and Control*. Nunnally, B. K., Turula, V. E. & Sitrin, R. D., eds. Heidelberg: Springer Berlin, 45–80 (2015).

[22] Zeng G (2021). Immunogenicity and safety of a third dose of CoronaVac, and immune persistence of a two-dose schedule, in healthy adults: interim results from two single-centre, double-blind, randomised, placebo-controlled phase 2 clinical trials. Lancet Infect. Dis..

[23] Vo HTM (2022). Robust and functional immune memory up to 9 months after SARS-CoV-2 infection: a Southeast Asian longitudinal cohort. Front. Immunol..

[24] Bates TA (2022). Vaccination before or after SARS-CoV-2 infection leads to robust humoral response and antibodies that effectively neutralize variants. Sci. Immunol..

[25] Wang Z (2021). Naturally enhanced neutralizing breadth against SARS-CoV-2 one year after infection. Nature.

[26] Zhou B (2022). A fourth dose of Omicron RBD vaccine enhances broad neutralization against SARS-CoV-2 variants including BA.1 and BA.2 in vaccinated mice. J. Med. Virol..

[27] Xia S (2022). Safety and immunogenicity of an inactivated COVID-19 vaccine, WIBP-CorV, in healthy children: interim analysis of a randomized, double-blind, controlled, phase 1/2 trial. Front. Immunol..

[28] Liu J (2021). Immunogenicity and safety of a 3-dose regimen of a SARS-CoV-2 inactivated vaccine in adults: a randomized, double-blind, placebo-controlled phase 2 trial. J. Infect. Dis..

[29] Medeiros-Ribeiro AC (2021). Immunogenicity and safety of the CoronaVac inactivated vaccine in patients with autoimmune rheumatic diseases: a phase 4 trial. Nat. Med..

[30] Stamatatos L (2021). mRNA vaccination boosts cross-variant neutralizing antibodies elicited by SARS-CoV-2 infection. Science.

[31] Mahase E (2022). Covid-19: what we know about the BA.4 and BA.5 omicron variants. BMJ.

[32] Garcia-Beltran WF (2021). COVID-19-neutralizing antibodies predict disease severity and survival. Cell.

[33] Grifoni A (2020). Targets of T cell responses to SARS-CoV-2 coronavirus in humans with COVID-19 disease and unexposed individuals. Cell.

[34] Lakshmanappa YS (2021). SARS-CoV-2 induces robust germinal center CD4 T follicular helper cell responses in rhesus macaques. Nat. Commun..

[35] Adeloye D (2021). The long-term sequelae of COVID-19: an international consensus on research priorities for patients with pre-existing and new-onset airways disease. Lancet Respir. Med..

[36] Mehandru S, Merad M (2022). Pathological sequelae of long-haul COVID. Nat. Immunol..

[37] Carreño JM (2022). Activity of convalescent and vaccine serum against SARS-CoV-2 Omicron. Nature.

[38] Turner JS (2021). SARS-CoV-2 mRNA vaccines induce persistent human germinal centre responses. Nature.

[39] Zhong D (2021). Durability of antibody levels after vaccination with mRNA SARS-CoV-2 vaccine in individuals with or without prior infection. JAMA.

[40] Evans JP (2022). Neutralizing antibody responses elicited by SARS-CoV-2 mRNA vaccination wane over time and are boosted by breakthrough infection. Sci. Transl. Med..

[41] Evans JP (2022). Neutralization of SARS-CoV-2 Omicron sub-lineages BA.1, BA.1.1, and BA.2. Cell Host Microbe.

[42] Neerukonda SN (2022). Characterization of entry pathways, species-specific angiotensin-converting enzyme 2 residues determining entry, and antibody neutralization evasion of omicron BA.1, BA.1.1, BA.2, and BA.3 variants. J. Virol..

[43] Han P (2022). Receptor binding and complex structures of human ACE2 to spike RBD from omicron and delta SARS-CoV-2. Cell.

[44] Zhao X (2022). Omicron SARS-CoV-2 neutralization from inactivated and ZF2001 vaccines. N. Engl. J. Med..

[45] Park JH (2022). Relationship between SARS-CoV-2 antibody titer and the severity of COVID-19. J. Microbiol. Immunol. Infect..

[46] Yamayoshi S (2021). Antibody titers against SARS-CoV-2 decline, but do not disappear for several months. EClinicalMedicine.

[47] Robbiani DF (2020). Convergent antibody responses to SARS-CoV-2 in convalescent individuals. Nature.

[48] Yang HS (2021). Association of age with SARS-CoV-2 antibody response. JAMA Netw. Open.

[49] Sureshchandra S (2021). Single-cell profiling of T and B cell repertoires following SARS-CoV-2 mRNA vaccine. JCI Insight.

[50] Zhang J (2021). One-year sustained cellular and humoral immunities of COVID-19 convalescents. Clin. Infect. Dis..

[51] Vikkurthi R (2022). Inactivated whole-virion vaccine BBV152/Covaxin elicits robust cellular immune memory to SARS-CoV-2 and variants of concern. Nat. Microbiol..

[52] Peluso MJ (2021). Long-term SARS-CoV-2-specific immune and inflammatory responses in individuals recovering from COVID-19 with and without post-acute symptoms. Cell Rep..

[53] Bastard P (2021). Autoantibodies neutralizing type I IFNs are present in ~4% of uninfected individuals over 70 years old and account for ~20% of COVID-19 deaths. Sci. Immunol..

[54] Jung JH (2021). SARS-CoV-2-specific T cell memory is sustained in COVID-19 convalescent patients for 10 months with successful development of stem cell-like memory T cells. Nat. Commun..

[55] Chen G (2019). Clinical and immunological features of severe and moderate coronavirus disease 2019. J. Clin. Invest.

[56] Weiskopf D (2020). Phenotype and kinetics of SARS-CoV-2-specific T cells in COVID-19 patients with acute respiratory distress syndrome. Sci. Immunol..

[57] Mateus J (2021). Low-dose mRNA-1273 COVID-19 vaccine generates durable memory enhanced by cross-reactive T cells. Science.

[58] Godeau D, Petit A, Richard I, Roquelaure Y, Descatha A (2021). Return-to-work, disabilities and occupational health in the age of COVID-19. Scand. J. Work Environ. Health.

[59] Tarke A (2022). SARS-CoV-2 vaccination induces immunological T cell memory able to cross-recognize variants from Alpha to Omicron. Cell.

[60] Yu D (2022). Potent anti-SARS-CoV-2 efficacy of COVID-19 hyperimmune globulin from vaccine-immunized plasma. Adv. Sci..

[61] Nie J (2019). Nipah pseudovirus system enables evaluation of vaccines in vitro and in vivo using non-BSL-4 facilities. Emerg. Microbes Infect..

[62] Peng Y (2020). Broad and strong memory CD4+ and CD8+ T cells induced by SARS-CoV-2 in UK convalescent individuals following COVID-19. Nat. Immunol..

