# Supplementary material for: COVID-19 vaccination boosts the potency and breadth of the immune response against SARS-CoV-2 among recovered patients in Wuhan
Source: Cell Discov. 2022 Dec 9;8:131. doi: 10.1038/s41421-022-00496-x (PMC9734167; doi:10.1038/s41421-022-00496-x)
Supplement: Supplementary file 1 — Supplementary information [file 41421_2022_496_MOESM1_ESM.pdf]

Supplementary Materials for

COVID-19 vaccination boosts the potency and breadth of the immune response against SARS-CoV-2 among recovered patients in Wuhan

Hong Liang[1,#], Xuanxuan Nian[2,3,#], Junzheng Wu[4,#], Dong Liu[1,#], Lu Feng[5], Jia Lu[2,3], Yan Peng[5], Zhijun Zhou[5], Tao Deng[2,3], Jing Liu[2,3], Deming Ji[5], Ran Qiu[2,3], Lianzhen Lin[5], Yan Zeng[2,3], Fei Xia[2,3], Yong Hu[5], Taojing Li[1], Kai Duan[2,3], Xinguo Li[2,3], Zejun Wang[2,3], Yong Zhang[1], Hang Zhang[1], Chen Zhu[5], Shang Wang[5], Xiao Wu[5], Xiang Wang[2,3], Yuwei Li[2,3], Shihe Huang[2,3], Min Mao[5], Huanhuan Guo[6], Yunkai Yang[7], Rui Jia[7], Jingwei Xufang[7], Xuewei Wang[7], Shuyan Liang[8], Zhixin Qiu[8], Juan Zhang[8], Yaling Ding[4], Chunyan Li[1], Jin Zhang[5], Daoxing Fu[1], Yanlin He[1,5], Dongbo Zhou[1], Cesheng Li[5,*], Jiayou Zhang[2,3,*], Ding Yu[1,4,*], Xiao-Ming Yang[2,7,*]

[1]Beijing Tiantan Biological Products Co., Ltd., 100024 Beijing, China

[2]National Engineering Technology Research Center for Combined Vaccines, 430207 Wuhan, China

[3]Wuhan Institute of Biological Products Co., Ltd., 430207 Wuhan, China

[4]Chengdu Rongsheng Pharmaceuticals Co., Ltd., 610041 Chengdu, China

[5]Sinopharm Wuhan Plasma-derived Biotherapies Co., Ltd., 430207 Wuhan, China

[6]Wuxue Wusheng Plasma Collection Center, 435499 Wuxue, China

[7]China National Biotec Group Company Limited, 100029 Beijing, China

[8]Wuhan Biobank Co., Ltd., 430075 Wuhan, China

[#]These authors contributed equally: Hong Liang, Xuanxuan Nian, Junzheng Wu, Dong Liu

***Corresponding authors**

Xiaoming Yang (yangxiaoming@sinopharm.com); Ding Yu (yuding1@sinopharm.com); Jiayou Zhang (tjzhjy@126.com); Cesheng Li (licesheng@sinopharm.com).

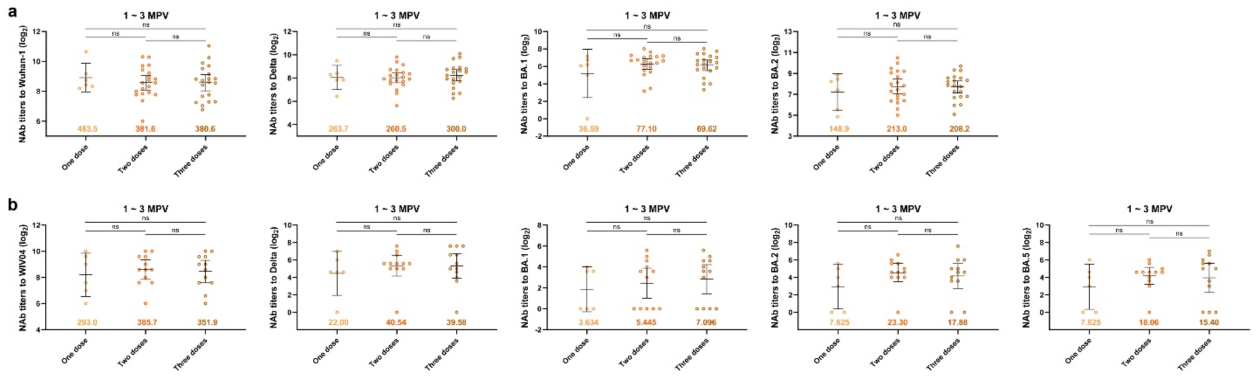

Supplementary Fig. S1. Effects of different doses of vaccine on neutralizing antibody (NAb) titers in the hybrid-immunity group. Comparison of the NAb titers against pseudotyped **(a)** and authentic **(b)** SARS-CoV-2 Wuhan-1, Delta, and Omicron BA.1 and BA.2 (and BA.5) strains in the hybrid immunity group at 1~3 months post-vaccination with one, two, or three vaccine doses. Error bars indicate the geometric mean titer (GMT) with the 95% confidence interval, and the colored numbers represent GMT values. Statistical significance was analyzed by the Kruskal–Wallis method. Wuhan-1, SARS-CoV-2 Wuhan-Hu-1. WIV04, SARS-CoV-2 nCoV-2019BetaCoV/Wuhan/WIV04/2019. MPV, months post-vaccination.

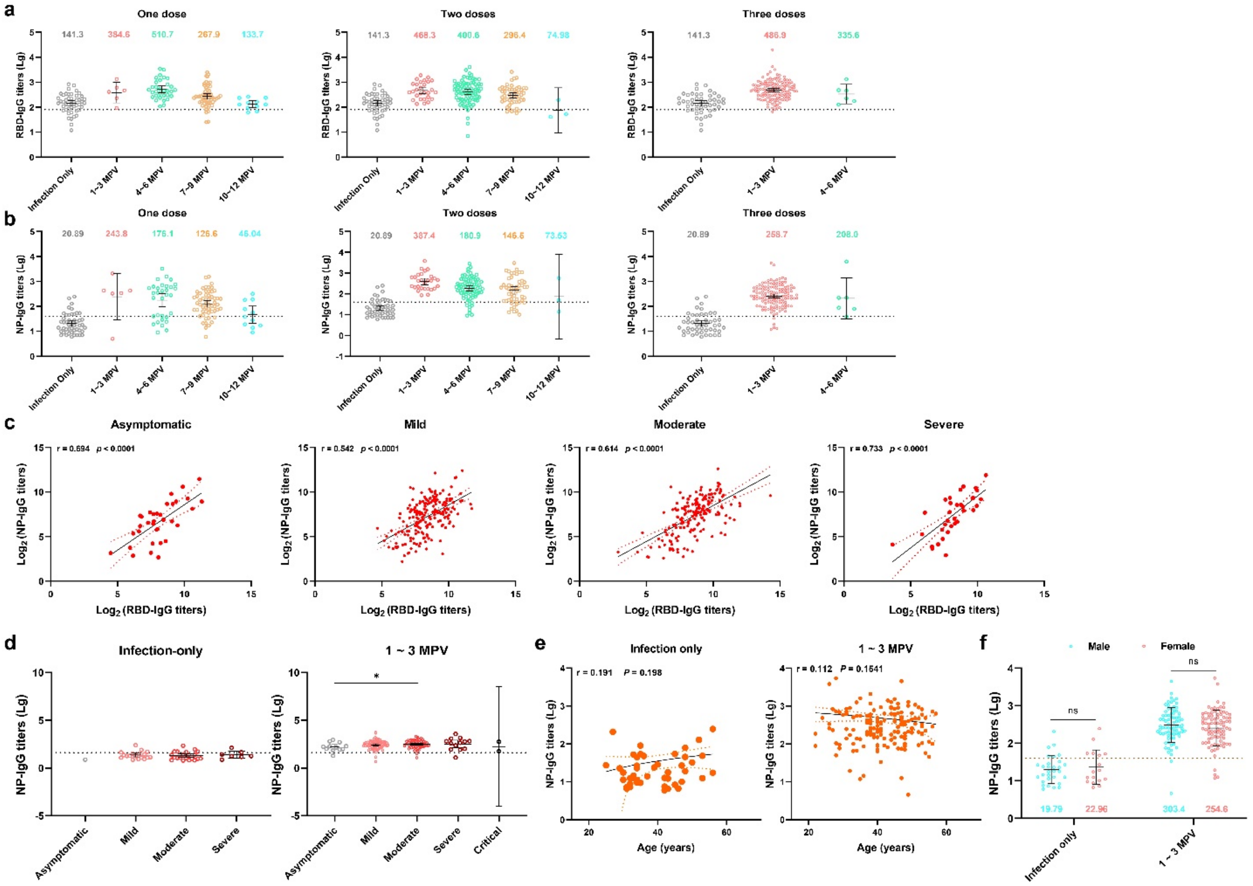

Supplementary Fig. S2. Effects of time and other factors on RBD-IgG and NP-IgG responses. **(a, b)** Decline of RBD-IgG (a) and NP-IgG (b) titers in the hybrid-immunity group individuals receiving one, two, or three vaccine doses over time, with the infection-only group serving as a control. **(c)** RBD-IgG titers are positively correlated with the NP-IgG titers across populations with different disease severities (samples included infection-only and hybrid-immunity groups). **(d-f)** No significant correlations were observed between NP-IgG titers and disease severity (d), age (e), or sex (f) in all groups. Error bars in (a), (b), (d) and (e) indicate the geometric mean titer (GMT) with the 95% confidence interval, where the colored numbers represent GMT values. Statistical significances in (d) were analyzed using the Kruskal–Wallis test, only groups that were significantly different are marked, and those in (f) were analyzed using a paired *t* test. Scatterplots

in (c) and (e) depict the simple linear fit with 95% confidence bands, together with the Spearman correlation coefficient and 2-tailed *P* value. MPV, months post-vaccination.

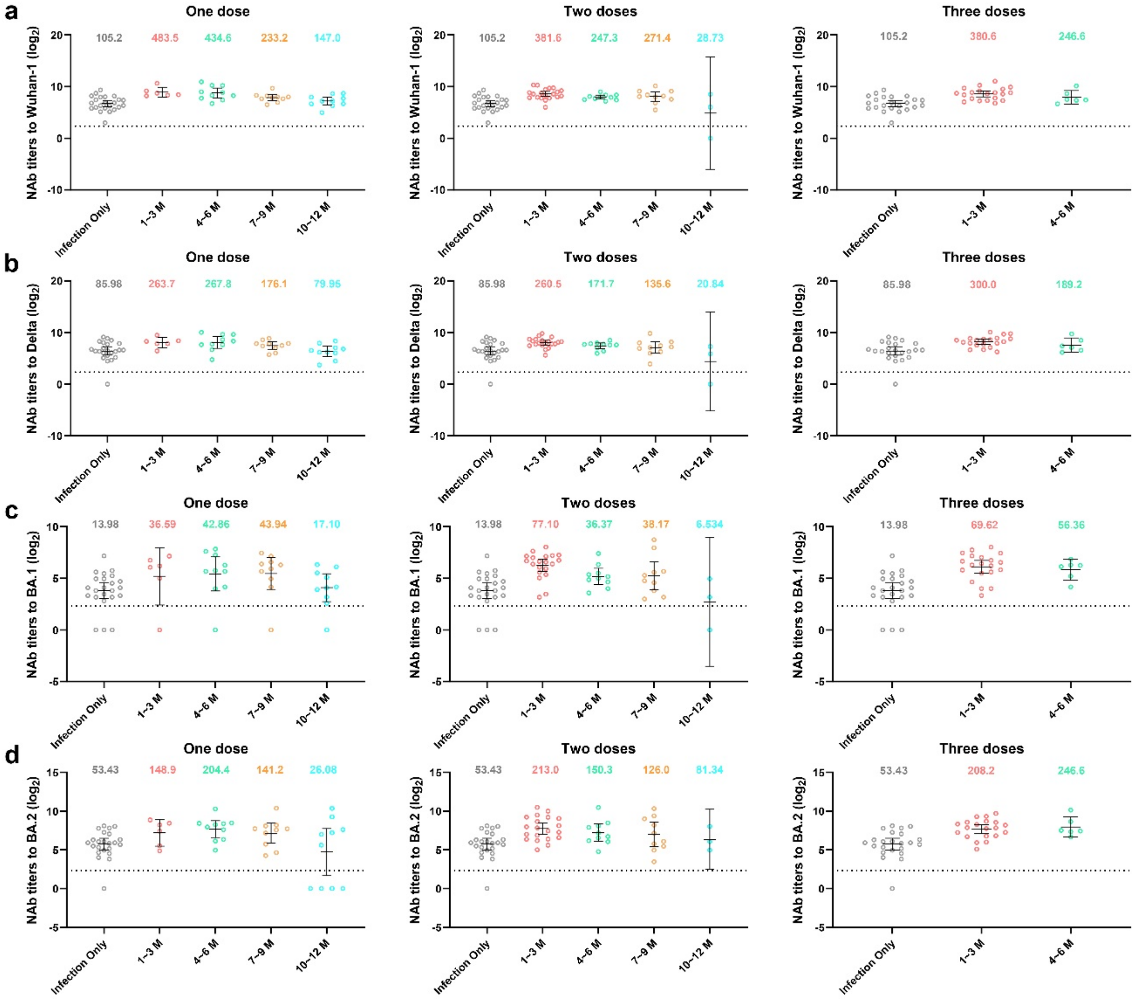

Supplementary Fig. S3. Effects of time after vaccination on neutralizing antibody (NAb) responses against the pseudotyped SARS-CoV-2 strains. (a-d) Decline in NAb titers against Wuhan-1 (a), Delta (b), and Omicron BA.1 (c), and BA.2 (d) in the hybrid-immunity group individuals receiving one, two, or three vaccine doses over time, with the infection-only group serving as a control. Wuhan-1, SARS-CoV-2 Wuhan-Hu-1. M, months post-vaccination

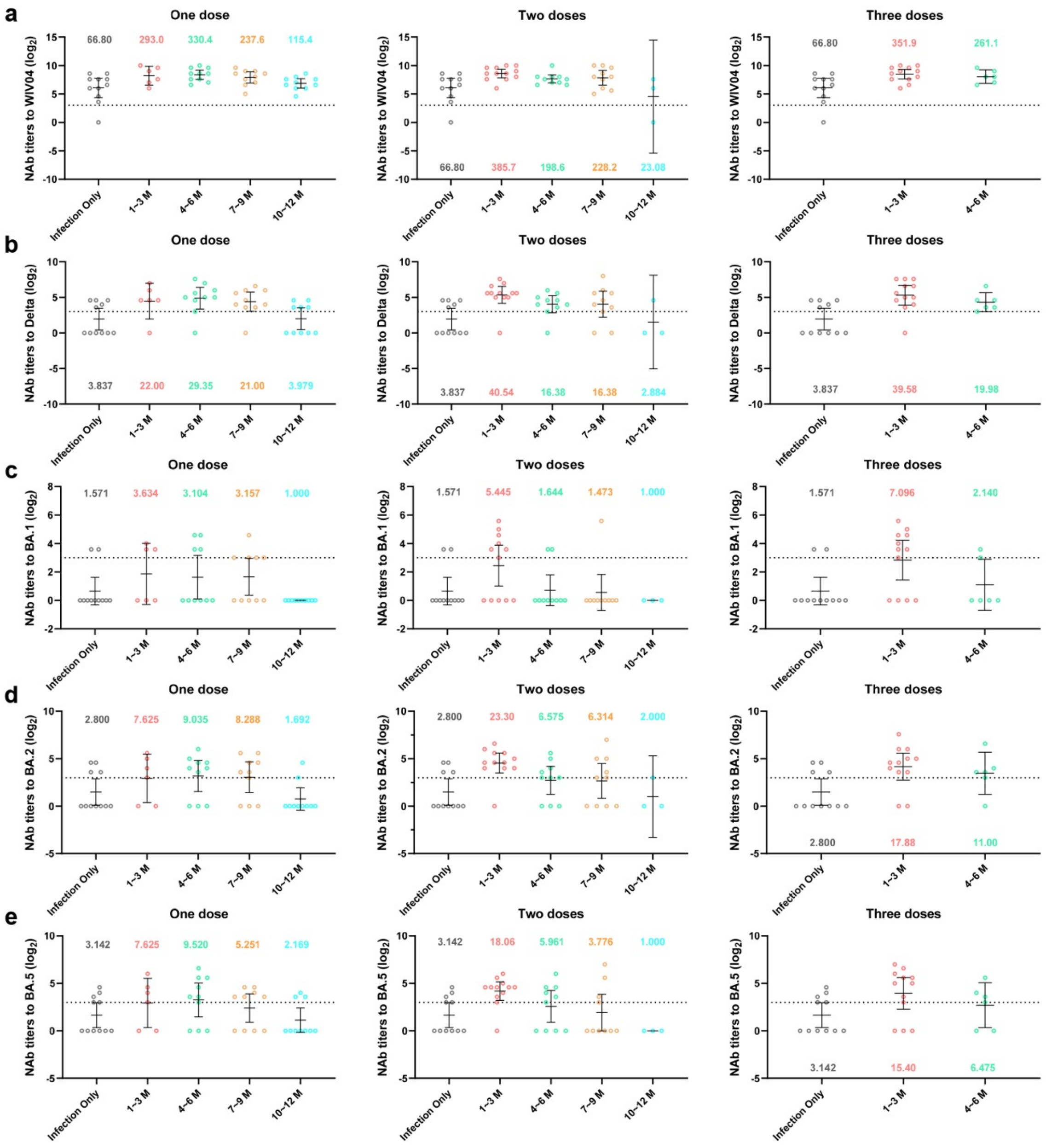

Supplementary Fig. S4. Effects of time after vaccination on neutralizing antibody (NAb) responses against authentic SARS-CoV-2 strains. (a-e) Decline in NAb titers against Wuhan-1 (a), Delta (b), and Omicron BA.1 (c), BA.2 (d), and BA.5 (e) in the hybrid-immunity group individuals receiving one, two, or three vaccine doses over time, with the infection-only group serving as a control. WIV04, SARS-CoV-2 nCoV-2019BetaCoV/Wuhan/WIV04/2019. M, months post-vaccination.

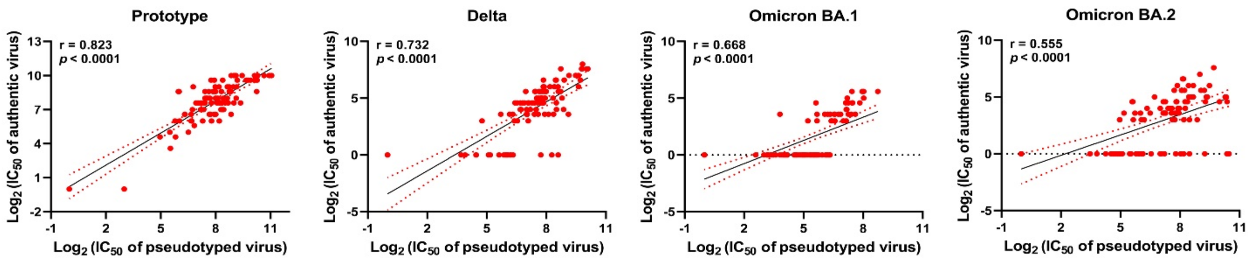

Supplementary Fig. S5. The neutralization efficiency of serum antibodies based on pseudotyped and authentic SARS-CoV-2 was positively correlated.

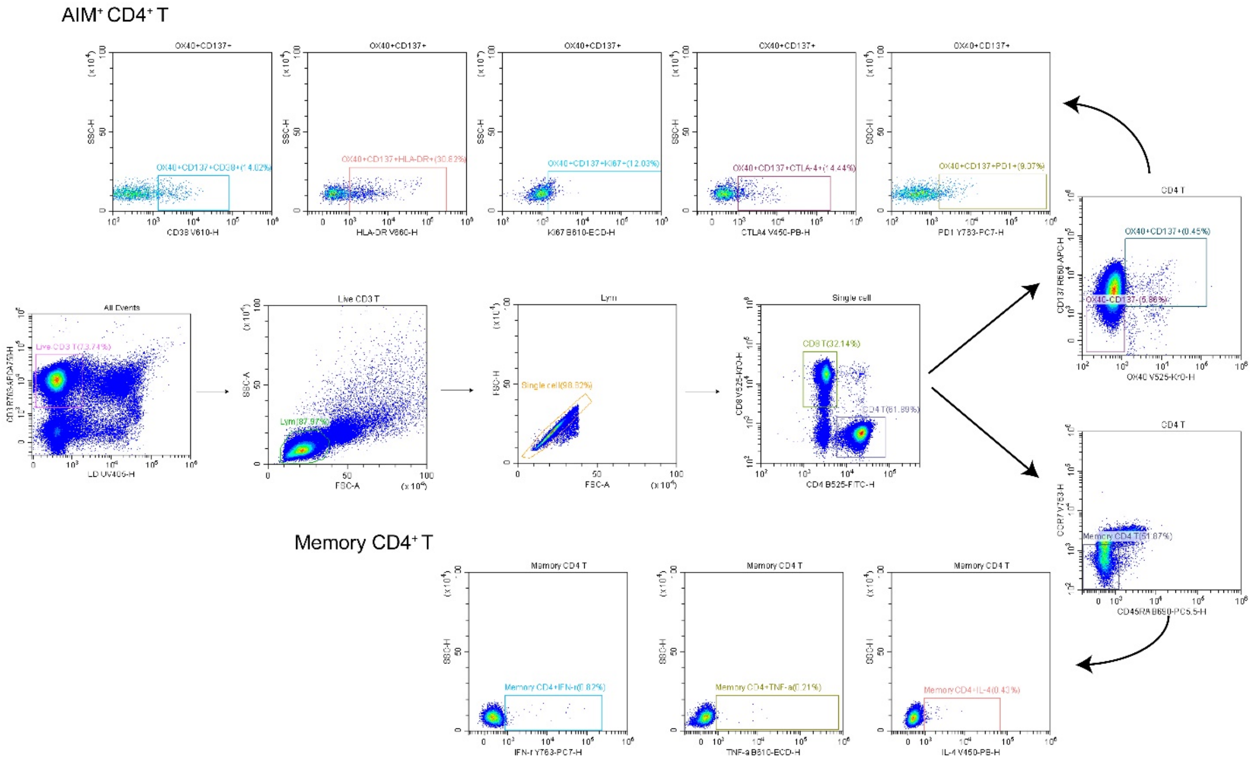

Supplementary Fig. S6. Representative example of the gating strategy for AIM and ICS assays.

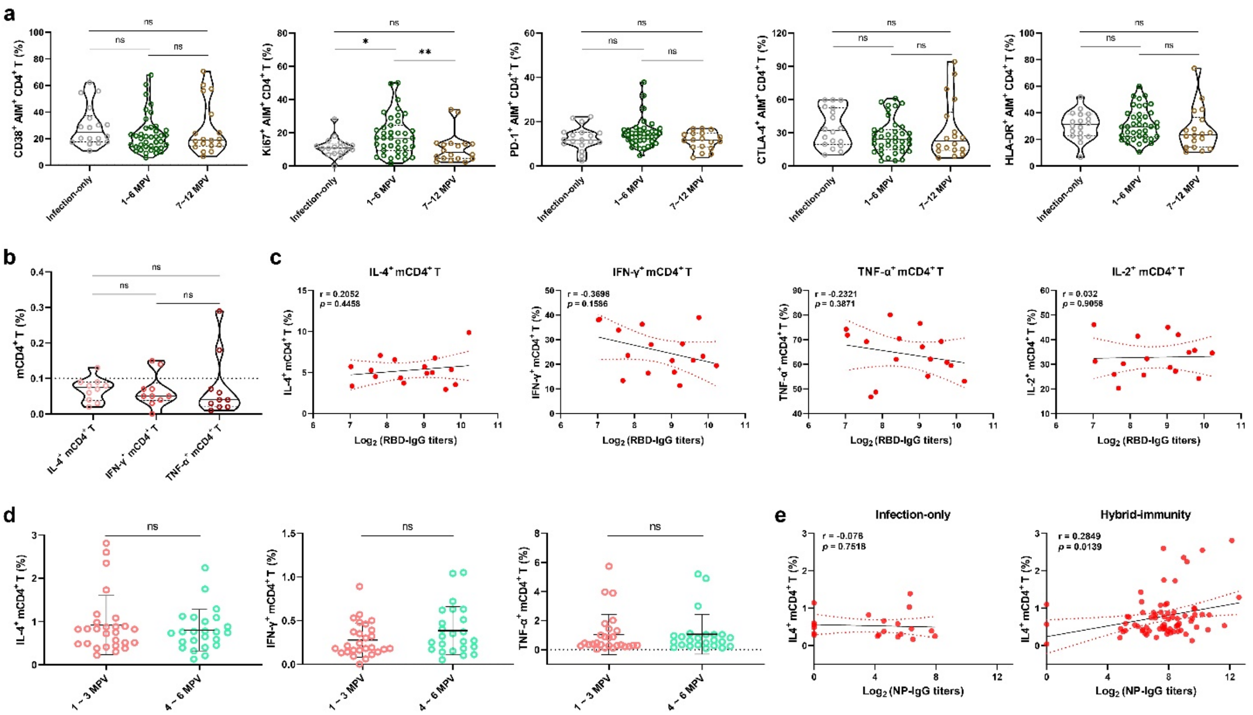

Supplementary Fig. S7. SARS-CoV-2-specific CD4$^+$ T cells in the peripheral blood of individuals for AIM and ICS assays. **(a)** Frequencies of Ki67$^+$ AIM$^+$ CD4$^+$ T, CD38$^+$ AIM$^+$ CD4$^+$ T, HLA-DR$^+$ AIM$^+$ CD4$^+$ T, CTLA-4$^+$ AIM$^+$ CD4$^+$ T, and PD-1$^+$ AIM$^+$ CD4$^+$ T cells following vaccination. **(b)** Frequencies of IL-4$^+$ mCD4$^+$, IFN-γ$^+$ mCD4$^+$, and TNF-α$^+$ mCD4$^+$ T cells without SARS-CoV-2 peptide pool stimulation, used as the negative control. **(c)** Frequencies of IL-4$^+$ mCD4$^+$, IFN-γ$^+$ mCD4$^+$, and TNF-α$^+$ mCD4$^+$ T cells detected following PMA stimulation, used as the positive control, and the correlation between the activated mCD4$^+$ T cell frequencies and the RBD-IgG titers. **(d)** Comparison of the frequencies of IL-4$^+$ mCD4$^+$, IFN-γ$^+$ mCD4$^+$, and TNF-α$^+$ mCD4$^+$ T cells 1-3 months and 4-6 months post-vaccination. **(e)** Correlation between the percentage of IL-4$^+$ mCD4$^+$ T cells and the magnitude of NP-IgG titers in the infection-only group and hybrid-immunity group. Statistical significances in (a-b) were analyzed using the Kruskal–Wallis test, and those in (d) were analyzed using a *t* test. Scatterplots in (c, e) depict the simple

linear fit with 95% confidence bands along with Spearman's correlation coefficient and two-tailed *P* value. MPV, months post-vaccination.

Supplementary Table S1. Samples for neutralizing antibody titer and flow cytometry analysis.

	RBD-IgG titers	Infection-only	Hybrid-immunity			
			**1~3 M**	**4~6 M**	**7~9 M**	**10~12 M**
**Pseudotyped virus, n**	Negative	6	0	1	2	3
	Low	10	14	9	11	10
	Moderate	7	27	12	5	0
	High	0	5	4	2	0
**Authentic virus, n***	Negative	3	0	1	2	3
	Low	8	9	9	11	10
	Moderate	0	16	12	5	0
	High	0	5	4	2	0
**AIM, n (a, b)**	Negative	3 (3[a], 0[b])	0 (0, 0)	0 (0, 0)	0 (0, 0)	0 (0, 0)
	Low	9 (9, 0)	9 (9, 0)	9 (8, 1)	14 (13, 1)	0 (0, 0)
	Moderate	5 (5, 0)	11 (8, 3)	10 (9, 1)	6 (5, 1)	0 (0, 0)
	High	1 (1, 0)	5 (3, 2)	3 (2, 1)	0 (0, 0)	0 (0, 0)
**ICS, n (a, b, c)**	Negative	2 (2[a], 0[b], 0[c])	0 (0, 0, 0)	0 (0, 0, 0)	0 (0, 0, 0)	0 (0, 0, 0)
	Low	12 (11, 0, 1)	12 (10, 0, 2)	15 (13, 1, 1)	18 (15, 1, 2)	0 (0, 0, 0)
	Moderate	8 (7, 0, 1)	20 (13, 3, 4)	13 (7, 1, 5)	8 (7, 1, 0)	0 (0, 0, 0)
	High	0 (0, 0, 0)	6 (4, 2, 0)	5 (4, 1, 0)	1 (1, 0, 0)	0 (0, 0, 0)

*, Samples of the hybrid-immunity group selected for authentic virus, all present in the pseudotyped virus group.

M, months post-vaccination. AIM, activation-induced marker. ICS, intracellular staining.

a, Experimental samples. b, Negative control samples. c, Positive control samples.

Supplementary Table S2. The variant-specific deletions and substitutions introduced in spike-pseudotyped SARS-CoV-2.

Pseudotyped viruses	Spike mutation
Wuhan-1	NA
Delta	T19R, G142D, F157del, R158del, L452R, T478K, D614G, P681R, D950N
Omicron BA.1	A67V, H69del, V70del, T95I, G142D, V143del, Y144del, Y145del, N211del, L212I, ins214EPE, G339D, S371L, S373P, S375F, K417N, N440K, G446S, S477N, T478K, E484A, Q493R, G496S, Q498R, N501Y, Y505H, T547K, D614G, H655Y, N679K, P681H, N764K, D796Y, N856K, Q954H, N969K, L981F
Omicron BA.2	T19I, L24del, P25del, P26del, A27S, G142D, V213G, G339D, S371F, S373P, S375F, T376A, D405N, R408S, K417N, N440K, S477N, T478K, E484A, Q493R, Q498R, N501Y, Y505H, D614G, H655Y, N679K, P681H, N764K, D796Y, Q954H, N969K

Wuhan-1, SARS-CoV-2 Wuhan-Hu-1. NA, not applicable.

Supplementary Table S3. The SARS-CoV-2-specific peptide pool used in this study.

Group (n)	Peptide	Position	Amino acid sequence
	S-34a	166–180	CTFEYVSQPFLMDLE
	S-39	191–205	EFVFKNIDGYFKIYS
	S-42	206–230	KHTPINLVRDLPQGF
	S-43	211–225	NLVRDLPQGFSALEP
	S-71	351–365	YAWNRKRISNCVADY
	S-77	381–395	GVSPTKLNDLCFTNV
	S-90	446–460	GGNYNYLYRLFRKSN
	S-91	451–465	YLYRLFRKSNLKPFE
**S (18)**	S-103	506–520	VVLSFELLHAPATVC
	S-145	721–735	SVTTEILPVSMTKTS
	S-150	746–760	STECSNLLLQYGSFC
	S-151a	751–765	NLLLQYGSFCTQLNR
	S-161	801–815	NFSQILPDPSKPSKR
	S-174a	866–880	TDEMIAQYTSALLAG
	S-235	1,171–1,185	GINASVVNIQKEIDR
	S-240	1,196–1,210	LIDLQELGKYEQYI
	S-242	1,206–1,220	YEQYIKWPWYIWLGF
	NP-12	82–95	DQIGYYRRATRRIR
	NP-15	101–113	MKDLSPRWYFYYL
	NP-16a	104–121	LSPRWYFYYLGTGPEAGL
**NP (10)**	NP-46	313–330	AFFGMSRIGMEVTPSGTW
	NP-47	321–338	GMEVTPSGTWLTYTGAIK
	NP-48	329–346	TWLTYTGAIKLDDKDPNF
	NP-50	344–361	PNFKDQVILLNKHIDAYK
	M19	133–150	LLESELVIGAVILRGHLR
	M-20a	141–158	GAVILRGHLRIAGHHLGR
	M-21	149–166	LRIAGHHLGRCDIKDLPK
**M (6)**	M-23	165–181	PKEITVATSRTLSYYKL
	M-24a	172–188	TSRTLSYYKLGASQRVA
	M-28	201–218	IGNYKLNTDHSSSSDNIA
	ORF3a-20	145–160	YFLCWHTNCYDYCIPY
	ORF3a-27	198–215	KDCVVLHSYFTSDYYQLY
	ORF3a-30	224–243	GVEHVTFFIYNKIVDEPEEH
**ORFs (7)**	ORF7a-2	9–25	LITLATCELYHYQECVR
	ORF7a-7	46–63	FHPLADNKFALTCFSTQF
	ORF7a-10	69–86	DGVKHVYQLRARSVSPKL

S, spike. NP, nucleocapsid protein. M, membrane. ORF, open reading frame.

Supplementary Table S4. The antibodies and other reagents used in ICS and AIM assays.

Antibodies	Identification	Vender	Catalog
**Panel 1: AIM antibodies**			
CD3	APC/Cyanine7 anti-human CD3	BioLegend	300426
CD4	FITC anti-human CD4	BioLegend	300506
CD8	Brilliant Violet 510™ anti-human CD8	BioLegend	344732
CD137	APC anti-human CD137 (4-1BB）	BioLegend	309810
OX40	Brilliant Violet 510™ anti-human CD134 (OX40)	BioLegend	350026
CD38	Brilliant Violet 605™ anti-human CD38	BioLegend	303532
Ki-67	PE/Dazzle™ 594 anti-human Ki-67	BioLegend	350534
HLA-DR	Brilliant Violet 650™ anti-human HLA-DR	BioLegend	307650
PD-1	PE/Cyanine7 anti-human CD279 (PD-1)	BioLegend	329918
CTLA-4	Brilliant Violet 421 anti-human CD152 (CTLA-4）	BioLegend	369606
Live/Dead	Fixable Viability Stain 440U	BD	566332
**Panel 2: ICS and immune cell lineage**			
CD3	APC/Cyanine7 anti-human CD3	BioLegend	300426
CD4	FITC anti-human CD4	BioLegend	300506
CD8	Brilliant Violet 510™ anti-human CD8	BioLegend	344732
CD45RA	PerCP/Cyanine5.5 anti-human CD45RA	BioLegend	304122
CCR7	BV 785 anti-human CD197 (CCR7) 353229	BioLegend	353229
IFN-γ	PE/Cyanine7 anti-human IFN-γ	BioLegend	502528
TNF-α	PE/Dazzle™ 594 anti-human TNF-α	BioLegend	502946
IL-4	Brilliant Violet 421™ anti-human IL-4	BioLegend	500826
Live/Dead	Fixable Viability Stain 440U	BD	566332
**Other reagents**			
	FBS	GIBICO	10099141
	RPMI 1640	GIBICO	A1049101
	BMSO	GIBICO	D2650
	Lymphoprep	STEMCELL	7861
	Cell Activation Cocktail (with Brefeldin A)	BioLegend	423304
	Brefeldin A Solution	BioLegend	420601
	Cell Staining Buffer	BioLegend	420201
	FOXP3 Fix/Perm Buffer Set	BioLegend	421403
	Fixation Buffer	BioLegend	420801
	Intracellular Staining Perm Wash Buffer (10X)	BioLegend	421002
